# Characterization of a Dopamine Transporter and Its Splice Variant Reveals Novel Features of Dopaminergic Regulation in the Honey Bee

**DOI:** 10.3389/fphys.2019.01375

**Published:** 2019-11-01

**Authors:** Vicky Zhang, Robert Kucharski, Courtney Landers, Sashika N. Richards, Stefan Bröer, Rowena E. Martin, Ryszard Maleszka

**Affiliations:** ^1^Research School of Biology, The Australian National University, Canberra, ACT, Australia; ^2^Faculty of Science and Technology, University of Canberra, Bruce, ACT, Australia

**Keywords:** biogenic amine, dopaminergic neurons, social behavior, heteromeric protein, spliced transporter, DNA methylation

## Abstract

Dopamine is an important neuromodulator involved in reward-processing, movement control, motivational responses, and other aspects of behavior in most animals. In honey bees (*Apis mellifera*), the dopaminergic system has been implicated in an elaborate pheromonal communication network between individuals and in the differentiation of females into reproductive (queen) and sterile (worker) castes. Here we have identified and characterized a honey bee dopamine transporter (AmDAT) and a splice variant lacking exon 3 (AmDATΔex3). Both transcripts are present in the adult brain and antennae as well as at lower levels within larvae and ovaries. When expressed separately in the *Xenopus* oocyte system, AmDAT localizes to the oocyte surface whereas the splice variant is retained at an internal membrane. Oocytes expressing AmDAT exhibit a 12-fold increase in the uptake of [^3^H]dopamine relative to non-injected oocytes, whereas the AmDATΔex3-expressing oocytes show no change in [^3^H]dopamine transport. Electrophysiological measurements of AmDAT activity revealed it to be a high-affinity, low-capacity transporter of dopamine. The transporter also recognizes noradrenaline as a major substrate and tyramine as a minor substrate, but does not transport octopamine, L-Dopa, or serotonin. Dopamine transport via AmDAT is inhibited by cocaine in a reversible manner, but is unaffected by octopamine. Co-expression of AmDAT and AmDATΔex3 in oocytes results in a substantial reduction in AmDAT-mediated transport, which was also detected as a significant decrease in the level of AmDAT protein. This down-regulatory effect is not attributable to competition with AmDATΔex3 for ER ribosomes, nor to a general inhibition of the oocyte’s translational machinery. *In vivo*, the expression of both transcripts shows a high level of inter-individual variability. Gene-focused, ultra-deep amplicon sequencing detected methylation of the *amdat* locus at ten 5′-C-phosphate-G-3′ dinucleotides (CpGs), but only in 5–10% of all reads in whole brains or antennae. These observations, together with the localization of the *amdat* transcript to a few clusters of dopaminergic neurons, imply that *amdat* methylation is positively linked to its transcription. Our findings suggest that multiple cellular mechanisms, including gene splicing and epigenomic communication systems, may be adopted to increase the potential of a conserved gene to contribute to lineage-specific behavioral outcomes.

## Introduction

Dopamine is a biogenic monoamine of special interest (Iversen and Iversen, 2007). In both vertebrates and invertebrates, it acts as a neurotransmitter in several distinct pathways operating across various regions of the brain (Torres et al., 2003). It has been implicated in motivation, reward, addiction, attention, salience, and movement control. Deficiencies in the neuronal dopaminergic system result in debilitating diseases in humans (Iversen and Iversen, 2007; Ashok et al., 2017; Mackie et al., 2018), and mutants of *Drosophila melanogaster* lacking the ability to synthesize dopamine show reduced activity, extended sleep-time, locomotor deficits, abnormalities in arousal and choice, and are hypophagic (Riemensperger et al., 2011). In insects, dopamine is also involved in post-mating pheromone responses and is a critical substrate for cuticle pigmentation and hardening (Cichewicz et al., 2017).

The dopaminergic system has been a focus of studies on the evolution of social behavior in honey bees (*Apis mellifera*) and other eusocial insects (Blenau and Erber, 1998; Scheiner et al., 2006; Beggs et al., 2007; Okada et al., 2015). Several aspects of reproduction, behavioral maturation, and social dominance in eusocial societies have been associated with biogenic amines (Harris and Woodring, 1995; Harano et al., 2005, 2008; Okada et al., 2015). In an evolutionary context, a compelling idea is that biogenic amines and their receptors have been co-opted to control physiology and behaviors in insects leading to the emergence of eusocial societies (Kamhi et al., 2017). Studies by Mercer and colleagues have revealed that dopamine plays a critical role in social communication via the peripheral modulation of worker bee responses to the queen mandibular pheromone (QMP) (Beggs et al., 2007; Vergoz et al., 2007). A plausible mechanism by which the queen bee influences the colony is via QMP components such as homovanillyl alcohol, which structurally resembles dopamine (Jarriault and Mercer, 2012). One hypothesized benefit to the bee society from this blocking effect is the neutralization of any unpleasant perception of the high levels of QMP. This idea is supported by the observation that only very young bees are attracted to the queen, whereas older nurses and foraging individuals are repelled by QMP (Jarriault and Mercer, 2012).

Like other neuromodulators, dopamine exerts its action via a molecular system composed of G protein-coupled receptors and transporters, the role of which is to control dopamine storage, release, and reuptake (Torres et al., 2003). In *Apis*, AmDOP1–3 have been characterized in detail and are known to be expressed in the brain and antennae (Blenau et al., 1998; Humphries et al., 2003; Mustard et al., 2003; Beggs et al., 2005; Beggs and Mercer, 2009). By contrast, nothing is known about the proteins responsible for dopamine transport in this species, or how these proteins regulate the timing and strength of neurotransmission as well as the pre-synaptic pool of dopamine. In other organisms, dopamine released into the synaptic cleft is deposited back into the surrounding cells primarily by transporters of the solute carrier 6 (SLC6) family (also known as the neurotransmitter:sodium symporter family, T.C. 2.A.22). A number of SLC6 proteins have been shown to transport one or more monoamines, including dopamine, serotonin, noradrenaline, octopamine, and tyramine (Bröer and Gether, 2012). Both the vertebrate and invertebrate nervous systems feature dopamine and serotonin, whereas noradrenaline is replaced in invertebrates with octopamine and tyramine (Roeder, 2005). The monoamine transporters of the SLC6 family typically display high affinities for their substrates (which are present at very low levels in the synapses) and become saturated at low monoamine concentrations (Bröer and Gether, 2012). Moreover, many of the insect and mammalian monoamine transporters are sensitive to cocaine, a neurotoxin that acts as an effective plant defense compound by disrupting motor control in herbivores, but which is rewarding and highly addictive in humans (Barron et al., 2009; Eriksen et al., 2009). Cocaine acts by binding reversibly to a site overlapping, but not identical to, the conserved substrate binding-site of monoamine transporters, thereby inhibiting the reuptake of released monoamines and prolonging their synaptic effect (Beuming et al., 2008). However, despite the substantial research efforts driven by the public health costs of cocaine, it is currently unclear which of the monoamine transporter(s) are responsible for mediating the effect of cocaine.

Dopamine and serotonin transporters from insects are convenient models that have already provided important insights into the action of cocaine, and of other drugs, on these neurotransmitter systems (Porzgen et al., 2001; Sandhu et al., 2002). Insect monoamine transporters are also important proteins in themselves given their potential to serve as novel targets for harnessing or controlling insects to achieve economic and/or human health gains (Caveney and Donly, 2002; Malutan et al., 2002). Here we have identified and characterized a honey bee dopamine transporter (AmDAT) as well as a novel splice variant (AmDATΔex3) of this protein. A broad array of assays and database resources were utilized to gain an understanding of (i) the structure, methylation, and transcription of the *amdat* gene, (ii) the functions of the AmDAT and AmDATΔex3 proteins, and (iii) the interactions of AmDAT with several monoamines and cocaine. Taken together, our findings reveal a complex picture for AmDAT and its splice variant, including novel properties that may play a role in animal social interactions. The insights presented here suggest that multiple levels of cellular regulation, including epigenomic modifications and alternative splicing, may be modulating AmDAT activity to generate complex phenotypic and behavioral outcomes. As such, this work provides a foundation for unraveling how these regulatory networks recruit relatively simple and highly conserved molecules, such as neurotransmitters, to perform lineage-specific roles (Miklos and Maleszka, 2011; Maleszka, 2016).

## Experimental Procedures

### Compounds Used in This Study

[^3^H]dopamine and [^3^H]hypoxanthine were purchased from PerkinElmer. Dopamine, octopamine, L-Dopa, tyramine, serotonin, noradrenaline and cocaine were purchased from Sigma-Aldrich. Solutions containing monoamines were prepared fresh prior to each experiment to prevent oxidation of the monoamines.

### Cloning of the Honey Bee DAT Gene and Other Molecular Methods

The strategy employed to clone the full-length coding regions of *amdat* and *amdatΔex3* is shown in Supplementary Figure S1. It involved adding a synthetic fragment to extend the missing 5-end of the longest clone recovered from the brain cDNA. Recombinant plasmids harvested from liquid bacterial cultures did not contain any non-synonymous polymorphisms in the *amdat* sequence (Supplementary Table S1), indicating that it was suitable for further *in vitro* characterization. Transcriptional profiling was undertaken by qPCR as described previously (Becker et al., 2016; Kucharski et al., 2016). Gene-focused DNA methylation analyses were performed using amplicons generated from bisulfite-converted brain and antennal DNAs followed by ultra-deep sequencing on Illumina MiSeq platform (Wedd et al., 2016). All experimental procedures, including honey bee collections, are detailed in the Supplementary Material.

### Generation of the Constructs for *Xenopus* Oocyte Expression

The coding sequence of the Emerald Green Fluorescent Protein (EmGFP) was amplified from the pJTI^TM^ R4 Dest CMV N-EmGFP pA vector (Invitrogen) and inserted into the oocyte expression vector pGEM-He-Juel. Sequences encoding versions of AmDAT and AmDATΔex3 tagged with the human influenza hemagglutinin (HA) epitope were synthesized by GenScript and inserted into pGEM-He-Juel. A HA-tag was inserted into the second extracellular loop of AmDAT via the introduction of the nucleotide sequence gcaggagcttatccatacgatgttcctgactatgcagcaggagct between positions 495–496 of the AmDAT coding sequence (resulting in a HA-tag – YPYDVPDYA – flanked by an “AGA” peptide spacer at each end). This insertion point was selected because the presence of a HA-tag at the equivalent position of human DAT (HsDAT) did not affect the protein’s transport properties, expression, and/or trafficking, nor did it interfere with the nearby *N*-glycosylation sites (Sorkina et al., 2006; Bolan et al., 2007; Eriksen et al., 2009; Rao et al., 2012). By contrast, the addition of three HA-tags to the N-terminus of HsDAT was found to greatly diminish its dopamine transport activity (Vecchio et al., 2014) and we observed complete suppression of dopamine transport activity when two HA-tags were added to the N-terminus of AmDAT (data not shown). The HA-tagged forms of AmDAT and AmDATΔex3 are hereon referred to as HA_EL__2_-AmDAT and HA_EL__2_-AmDATΔex3, respectively.

### Preparation of cRNA

The plasmids were linearized with *Not*I or *Sal*I (ThermoFisher Scientific) and 5′-capped complementary RNA (cRNA) was transcribed *in vitro* using the mMessage mMachine T7 kit (Ambion) and then purified with the MEGAclear kit (Ambion). The samples were adjusted to the desired cRNA concentration using RNase-free elution buffer (Ambion) and the quality of the cRNA assessed via agarose gel electrophoresis.

### Harvest, Preparation, and Microinjection of *Xenopus* Oocytes

Oocytes were harvested and prepared as described in full elsewhere (van Schalkwyk et al., 2016). Briefly, sections of ovary were harvested from adult female frogs (purchased from NASCO, United States) via a minor surgical procedure and single, de-folliculated oocytes were prepared using a mix of collagenase A (Roche) and collagenase D (Roche). Stage V–VI oocytes were microinjected with cRNA (10 ng per oocyte) encoding AmDAT, AmDATΔex3, HA_EL__2_-AmDAT, or HA_EL__2_-AmDATΔex3. For the expression of EmGFP and the *Plasmodium falciparum* nucleoside transporter 1 (PfNT1), 15.4 fmol of cRNA was injected (equating to 5 ng of EmGFP cRNA and 7.6 ng of PfNT1 cRNA). The oocytes were stored at 16–18°C in OR2^+^ buffer (82.5 mM NaCl, 2.5 mM KCl, 1 mM MgCl_2_, 1 mM Na_2_HPO_4_, 5 mM HEPES, 1 mM CaCl_2_, and 50 μg/mL penicillin-streptomycin; pH 7.8).

### Immunofluorescence Analysis

Oocytes expressing HA_EL__2_-AmDAT or HA_EL__2_-AmDATΔex3 were fixed and labeled with antibodies 3 days post-cRNA-injection using a protocol detailed elsewhere (Richards et al., 2016). A mouse anti-HA antibody (Sigma, cat. no. H9658) and an Alexa Fluor 488 donkey anti-mouse antibody (Molecular Probes, cat no. A21202) were used at concentrations of 1:100 and 1:500, respectively. At least two independent experiments were performed (on oocytes from different frogs) for each oocyte type, within which slices were examined from a minimum of three oocytes.

### Western Blot Analyses

The semi-quantification of HA_EL__2_-AmDAT and HA_EL__2_-AmDATΔex3 protein levels in preparations of oocyte membranes was carried out using a protocol described in detail elsewhere (Summers et al., 2014) with the following minor modification: the final protein pellet was solubilized in 20 μL of a solution comprising 3 M urea, 100 mM DTT, 1% (v/v) SDS, 5 mM Tris–HCl (pH 7.6), 2.5 mM NaCl, 0.25% (v/v) Triton X-100, and 32.5% (v/v) NuPage sample loading buffer (Life Technologies). The levels of EmGFP protein were semi-quantified in extracts prepared from whole oocytes using a method outlined elsewhere (Marchetti et al., 2015), with the following minor modification: after pelleting the oocyte yolk, a sample of the supernatant was added to NuPage sample loading buffer (final concentration of 25% v/v) supplemented with 10 mM DTT. The protein samples prepared from oocyte membranes (HA_EL__2_-AmDAT and HA_EL__2_-AmDATΔex3) or whole oocytes (EmGFP) were then separated on a 4–12% Bis-Tris SDS-polyacrylamide gel (Life Technologies) and transferred to a Protran 0.45 μm nitrocellulose membrane (Amersham, GE Healthcare Life Sciences). The membranes were probed with a mouse anti-HA antibody (concentration of 1:2,000–1:4,000; Sigma-Aldrich, cat. no. H9658) or a mouse anti-GFP antibody (concentration of 1:3,000; Invitrogen, cat. no. MA5-15256), followed by a horseradish peroxidase-conjugated goat anti-mouse antibody (1:10,000; Life Technologies, cat. no. 626520). The protein bands were detected by chemiluminescence (Pierce), quantified using the Image J software (Schneider et al., 2012), and expressed as a percentage of the protein band intensity measured for the relevant control sample (i.e., oocytes expressing only HA_EL__2_-AmDAT, HA_EL__2_-AmDATΔex3, or EmGFP). Total protein staining was used to evaluate sample loading and efficiency of transfer; the membranes were rinsed with ultrapure water, stained with the MemCode^TM^ reversible protein stain kit (Pierce), and destained with ultrapure water. Densitometric analysis was performed using Image Studio Lite version 5.2 software (LI-COR). At least three independent experiments were performed (using oocytes from different frogs), and within each experiment the measurements were averaged from two independent replicates.

### Radioisotope Transport Assays

The uptake into oocytes of [^3^H]dopamine (0.14 μM; 21.2 Ci/mmol) and [^3^H]hypoxanthine (1.5 μM; 30 Ci/mmol) was measured 1–3 days post-cRNA-injection using a protocol described previously (Richards et al., 2016). The [^3^H]dopamine transport assays were conducted over 15 min at 27.5°C and in the presence of 0.86 μM unlabeled dopamine. The [^3^H]hypoxanthine transport assays were performed over 30 min at 27.5°C. The reaction buffer was either ND96 pH 6.0 ([^3^H]hypoxanthine transport assays) or ND96 pH 7.4 ([^3^H]dopamine transport assays) and contained 96 mM NaCl, 2 mM KCl, 1 mM MgCl_2_, 1.8 mM CaCl_2_, and 10 mM Tris–base supplemented with either 10 mM MES (pH 6.0 buffer) or 10 mM HEPES (pH 7.4 buffer). In all cases, at least three independent experiments were performed (using oocytes from different frogs), and within each experiment the measurements were made from 10 oocytes per treatment.

### Electrophysiological Recordings

Electrophysiological recordings were conducted 3–5 days post-cRNA-injection as described previously (Bröer, 2003; Böhmer et al., 2005). Briefly, all steady-state recordings were made with an Axon Geneclamp 500B amplifier (Axon Instruments). Voltage clamp was routinely set to –50 mV, and data were sampled at 3 Hz using pClamp 8.2 software (Axon Instruments). Oocytes were chosen for recording when they had a resting membrane potential –25 mV < *V*_*m*_ < –35 mV. ND96 pH 7.4 buffer (96 mM NaCl, 2 mM KCl, 1.8 mM MgCl_2_, 1 mM CaCl_2_ and 5 mM HEPES, titrated to pH 7.4 with NaOH) was used as the control solution for all electrophysiological recordings, unless indicated otherwise. To measure currents induced by various monoamines of interest, oocytes expressing AmDAT were superfused with ND96 pH 7.4 buffer containing a monoamine at a final concentration of 100 μM. After currents reached a plateau, superfusion was switched back to ND96 to observe reversibility of the currents. At least two independent experiments were performed (using oocytes from different frogs), and within each experiment the measurements were made from 8–11 oocytes per treatment.

Each datapoint or column in the Figures represents the activity (mean ± SEM.) for eight to eleven AmDAT-expressing oocytes. For all experiments bar octopamine inhibition, recordings were conducted over 2 weeks, using oocytes prepared from two different females, and the resulting data were pooled for individual experiments. All raw values were normalized to the first dopamine current in a recording.

### Measurements of EmGFP Fluorescence

The fluorescence intensity of oocytes expressing EmGFP was measured 1–3 days post-cRNA-injection. The oocytes were transferred to separate wells of a clear 96-well plate (Corning) and lysed in 50 μL of 20 mM Tris–HCl (pH 7.6) supplemented with the cOmplete^TM^ EDTA-free protease inhibitor cocktail (Roche). The fluorescence intensity was measured with a TECAN Infinite M1000 PRO plate reader. The excitation and emission wavelengths were 487 and 509 nm, respectively. In all cases, at least three independent experiments were performed (using oocytes from different frogs), and within each experiment the measurements were made from ten oocytes per treatment.

### Statistics

Statistical comparisons were made using one-way analysis of variance (ANOVA) in conjunction with Tukey’s multiple comparisons test. A difference was considered statistically significant if *p* < 0.05. Kinetic constants were derived from the electrophysiology data by non-linear curve fitting to the allosteric sigmoidal equation provided by Prism 5 (GraphPad).

## Results

### Computational Identification of a Putative Dopamine Transporter in the Honey Bee Genomic and Transcriptomic Databases

**FIGURE 1:**
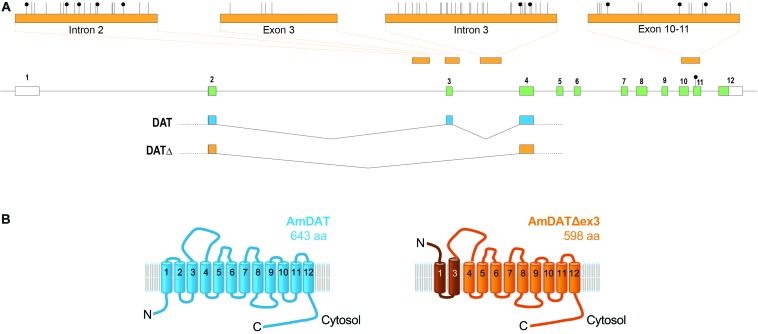
The honey bee dopamine transporter (AmDAT). **(A)** *amdat* gene model showing the exon and intron junctions of the full-length transcript and its spliced variant, the positions of methylated CpGs (black lollipops), and the regions of four amplicons (top) used for methylation analysis (see the main text and Supplementary Material for more details). The only methylated CpG uncovered by whole-genome methylomics is in exon 11. Protein coding exons are shown in green. **(B)** The predicted topologies of AmDAT (643 residues; shown in blue) and its splice variant AmDATΔex3 (598 residues; shown in orange). AmDATΔex3 lacks transmembrane domain 2 (TMD2) as well as the following cytosolic loop, resulting in an elongated TMD3. The affected region is shaded dark orange. The orientation of the N-terminus and TMD1 are predicted by TMpred and SPOCTOPUS to be inverted relative to the full-length protein.

We used the genomic assemblies and transcriptomic datasets available for the honey bee to extract all putative transporters belonging to the SLC6 family. The honey bee genome appears to encode 14 members of this family, including a protein (designated GB40867 in genome assembly V.4.5) that shows a high level of sequence similarity to the *Drosophil*a *melanogaster* dopamine transporter (DmDAT; Supplementary Table S2). The *amdat* gene model is shown in Figure 1A. AmDAT shows 71% identity and 85% similarity to DmDAT, and 50% identity and 71% similarity to the human noradrenaline/dopamine transporters.

The expression of *amdat* in different situations was evaluated by compiling a number of *Apis mellifera* transcriptomic databases. Consistent with its putative role in the reuptake of dopamine transport from the synaptic cleft, *amdat* is expressed in brains or heads of both adult and larval stages as well as in adult antennae. Transcripts are also present in libraries derived from other tissues, including queen ovaries and various glands (Supplementary Table S3).

Importantly, we detected an alternatively spliced transcript of *amdat* in libraries derived from honey bee brains. The transcript is missing exon 3, but maintains the reading frame of the coding sequence. The resulting protein, designated AmDATΔex3, would lack transmembrane domain 2 (TMD2) as well as the following linker region, and may consequently possess an elongated TMD3 (Figure 1B). Furthermore, the orientation of the N-terminus and TMD1 of AmDATΔex3 is predicted by TMpred and SPOCTOPUS (Hofmann and Stoffel, 1993; Viklund et al., 2008) to be inverted relative to these segments of the full-length protein. Given its significantly deformed topology, it seemed unlikely that AmDATΔex3 would function as a transporter. Nevertheless, its presence in several transcript libraries derived from honey bees collected in different geographical areas suggests that it serves a physiological role. All NCBI datasets in which *amdatΔex3* has been found are listed in Supplementary Table S3. In addition to brain, antennae, and ovaries, *amdatΔex3* is present in several glands.

### Expression of AmDAT and AmDATΔex3 in *Xenopus* Oocytes

**FIGURE 2:**
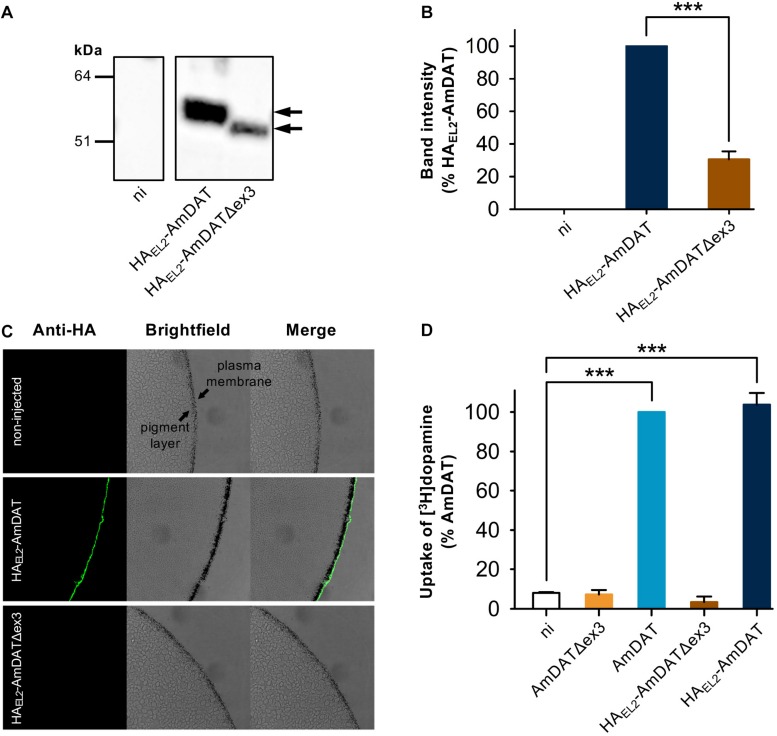
Heterologous expression of AmDAT and AmDATΔex3 in *Xenopus* oocytes. **(A)** Detection of HA_EL__2_-AmDAT (∼55 kDa band indicated by black arrow) and HA_EL__2_-AmDATΔex3 (∼50 kDa band indicated by black arrow) in oocyte membrane preparations. The samples were prepared on day 3 post-cRNA-injection, separated on a SDS-polyacrylamide gel, and probed with a mouse anti-HA antibody. A preparation of membrane protein from non-injected oocytes (ni) probed with the anti-HA antibody did not produce a band within this region. The image is representative of >3 independent experiments (performed using oocytes from different frogs). **(B)** Semi-quantification of the HA_EL__2_-AmDAT and HA_EL__2_-AmDATΔex3 proteins in the oocyte membrane. The intensity of the HA_EL__2_-AmDATΔex3 band was expressed as a percentage of the band measured for HA_EL__2_-AmDAT. The data are shown as the mean + SEM from six independent experiments (performed using oocytes from different frogs), within which measurements were averaged from two replicates. **(C)** Immunofluorescence microscopy of *Xenopus* oocytes expressing HA_EL__2_-AmDAT or HA_EL__2_-AmDATΔex3. The oocyte plasma membrane lies over a band of granules known as the “pigment layer”. This layer in turn surrounds a cytoplasm packed with yolk sacs and small endosomal- and lysosomal-type organelles. The expression of HA_EL__2_-AmDAT resulted in a fluorescent band external to the pigment layer, consistent with this protein being present in the oocyte plasma membrane. A fluorescent band was not detected in non-injected oocytes or in oocytes expressing HA_EL__2_-AmDATΔex3, suggesting that the splice variant is retained at an intracellular membrane. The images are representative of at least two independent experiments (performed using oocytes from different frogs), within which images were obtained from a minimum of 3 oocytes per oocyte type. **(D)** Transport of dopamine via AmDAT and HA_EL__2_-AmDAT. Measurements of [^3^H]dopamine uptake by oocytes expressing the non-tagged or HA-tagged forms of AmDAT or AmDATΔex3 were performed on day 3 post-cRNA-injection. Non-injected oocytes (ni) were included as the negative control. Dopamine uptake was expressed as a percentage of that measured in oocytes expressing AmDAT. The data are the mean + SEM of 3–4 independent experiments (performed using oocytes from different frogs), within which measurements were made from 10 oocytes per treatment. The capacities of HA_EL__2_-AmDAT and HA_EL__2_-AmDATΔex3 for dopamine transport did not differ significantly from those of their non-tagged counterparts (*p* > 0.05), consistent with the HA-tag exerting little or no effect upon the functions of these proteins. ^∗∗∗^denotes a significant difference (*p* < 0.001) between the indicated treatments (one-way ANOVA).

We used the *Xenopus* oocyte expression system to assess the transport activities of AmDAT and AmDATΔex3. In both cases, the expression of the desired protein was verified by injecting oocytes with cRNA encoding a hemagglutinin (HA)-tagged version of the protein (HA_EL__2_-AmDAT and HA_EL__2_-AmDATΔex3, respectively). Semi-quantitative western blot analyses of oocyte membrane preparations with an anti-HA antibody detected a band corresponding to ∼55 kDa for HA_EL__2_-AmDAT [predicted size of 72 kDa, noting that the binding of SDS tends to accelerate the migration of membrane transport proteins in SDS-PAGE (Rath et al., 2009)] and a band corresponding to ∼50 kDa for HA_EL__2_-AmDATΔex3 (predicted size of 67 kDa) (Figure 2A). These analyses also indicated that HA_EL__2_-AmDATΔex3 was present at much lower levels than HA_EL__2_-AmDAT in the oocyte membrane preparations (*p* < 0.001; Figure 2B). Total protein staining of the nitrocellulose membranes confirmed this observation was not due to uneven sample loading and/or uneven transferral of proteins from the SDS-PAGE gel (Supplementary Figure S2).

The presence of HA_EL__2_-AmDAT in the oocyte plasma membrane was confirmed with an immunofluorescence microscopy assay (Figure 2C). By contrast, HA_EL__2_-AmDATΔex3 was not detected at the oocyte surface, nor was it evident immediately below the oocyte plasma membrane. Given that antibodies will not penetrate into the yolk-sac laden interior of the oocyte, this assay could not ascertain the localization of the splice variant. However, the detection of HA_EL__2_-AmDATΔex3 in oocyte membrane preparations (Figures 2A,B), and its absence from the oocyte surface, suggests that the splice variant is retained in an intracellular membrane.

The capacities of HA_EL__2_-AmDAT and HA_EL__2_-AmDATΔex3, as well as of the non-tagged proteins, to transport dopamine were tested with a radioisotope uptake assay. The direction of [^3^H]dopamine transport in these experiments was from the extracellular medium (pH 7.4) into the oocyte cytosol, which corresponds to the reuptake of dopamine from the presynaptic cleft into the cytosol of dopaminergic neurons. The uptake of [^3^H]dopamine by non-injected oocytes was very low; this represented the background level of [^3^H]dopamine accumulation (Figure 2D). Oocytes expressing AmDAT or HA_EL__2_-AmDAT showed a marked increase in [^3^H]dopamine transport relative to non-injected oocytes (12.6 ± 0.6- and 12.3 ± 0.5-fold increases, respectively; mean ± SEM, *n* = 4–7; *p* < 0.001), with the inclusion of the HA-tag having no effect on the protein’s ability to mediate dopamine uptake (*p* > 0.05). By contrast, neither AmDATΔex3 nor HA_EL__2_-AmDATΔex3 caused an increase in the accumulation of [^3^H]dopamine within oocytes (Figure 2D).

### Transport Properties of AmDAT in *Xenopus* Oocytes

**FIGURE 3:**
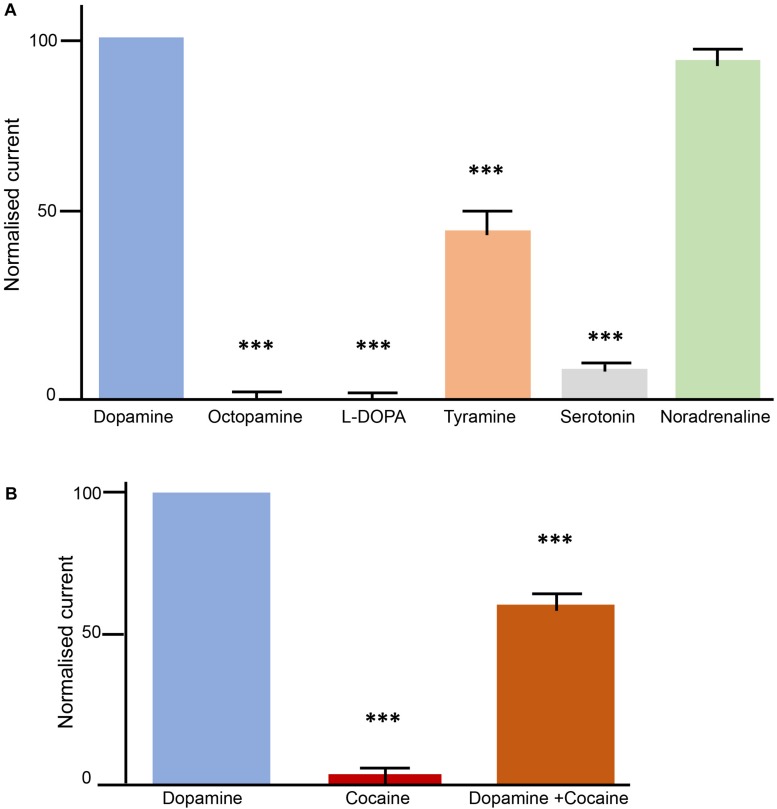
Transport properties of AmDAT in *Xenopus* oocytes. Measurements were conducted with oocytes expressing AmDAT on day 3 post-cRNA-injection. The oocytes were held at a membrane potential of –50 mV and superfused with ND96 (pH 7.4) or ND96 (pH 7.4) containing different monoamines at a final concentration of 100 μM. Each bar represents the mean and SEM of *n* = 11 oocytes (using oocytes from at least two different frogs). The data were normalised to the current induced by 100 μM dopamine. ^∗∗∗^denotes a significant difference (*p* < 0.001) from the dopamine current at 100 μM (one-way ANOVA). **(A)** Substrate specificity of AmDAT. **(B)** Inhibition of AmDAT by cocaine.

The successful expression of AmDAT at the oocyte surface enabled the use of electrophysiology to investigate the protein’s transport properties. The substrate-specificity of AmDAT was investigated by superfusing oocytes with seven potential substrates in the following order: dopamine, octopamine, L-Dopa, dopamine, tyramine, serotonin, noradrenaline, and dopamine. All currents are expressed as a percentage of the first dopamine current. The currents induced with octopamine (0.5 ± 2.5; mean ± SEM, *n* = 11; *p* < 0.001), L-Dopa (–0.9 ± 2.5; *n* = 10; *p* < 0.001), serotonin (6.6 ± 1.8; *n* = 11; *p* < 0.001), and tyramine (44.9 ± 7.8; *n* = 9; *p* < 0.001) were significantly smaller than the currents induced with dopamine (Figure 3A). By contrast, the current induced with noradrenaline (92.4 ± 4.8; *n* = 10; *p* > 0.05) was not significantly smaller than that induced by dopamine. These results indicate that dopamine and noradrenaline are major substrates of AmDAT, with tyramine being a minor substrate. Octopamine, L-Dopa, and serotonin do not appear to be substrates of AmDAT.

The kinetics of dopamine transport via AmDAT were determined by superfusing oocytes with a series of dopamine solutions of different concentrations. The superfusions were first performed in decreasing order of concentration, after which the same series was performed in reverse (and with the same oocyte). The relationship between the dopamine-induced current and the dopamine concentration is shown in Supplementary Figure S3A. Non-linear regression was used to fit an allosteric sigmoidal equation (a slightly modified version of the Hill equation) to the normalized data. The values obtained for K_0__.__5_ (5.6 ± 3 μM) and the Hill coefficient n (2.4 ± 0.2) indicate, respectively, that AmDAT is a high-affinity, low-capacity transporter of dopamine and that it forms a dimer and operates in a cooperative manner.

### AmDAT Is Inhibited by Cocaine, but Not by Octopamine

The interactions of cocaine and octopamine with AmDAT were investigated by superfusing oocytes with solutions of dopamine, the test compound, or a mixture of both dopamine and the test compound. The pattern applied in these experiments (illustrated in Supplementary Figure S3B) was designed to ascertain whether the test compound was a substrate or an inhibitor of AmDAT, and if the latter, whether binding to AmDAT was reversible. All currents are expressed as a percentage of the first dopamine current. Cocaine did not produce a significant current in Am-DAT expressing oocytes (Figure 3B); the mean ± SEM value for cocaine was 3.4 ± 1.7 (*n* = 11; *p* < 0.001). The presence of cocaine decreased the current induced by dopamine to 61.6 ± 4.9 (*n* = 10; *p* < 0.001), whereas octopamine was without effect (data not shown). AmDAT-expressing oocytes that had been superfused with cocaine or a dopamine + cocaine mix retained their ability to produce dopamine-induced currents (*n* = 10; *p* < 0.001), consistent with cocaine binding to AmDAT in a reversible manner (Supplementary Figure S3B). Together, the data indicate that AmDAT does not interact with octopamine, and that cocaine is an inhibitor, but not a substrate, of the transporter.

### Characterization of AmDATΔex3 in *Xenopus* Oocytes

#### AmDATΔex3 Reduces Dopamine Transport via AmDAT

**FIGURE 4:**
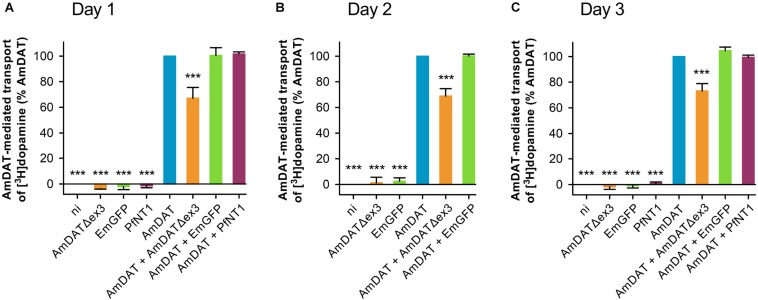
AmDATΔex3 downregulates the AmDAT-mediated uptake of dopamine in *Xenopus* oocytes. Measurements of [^3^H]dopamine transport were performed on **(A)** day 1, **(B)** day 2, and **(C)** day 3 post-cRNA-injection. Non-injected oocytes (ni) were included as a negative control, EmGFP and PfNT1 were included as co-injection controls, and oocytes expressing AmDAT served as the positive control. Dopamine uptake was expressed as a percentage of that measured in the AmDAT-expressing oocytes. The component of [^3^H]dopamine transport attributable to AmDAT was calculated by subtracting the background level of accumulation (i.e., dopamine uptake in non-injected oocytes) from that measured for each of the other oocyte types (the total levels of [^3^H]dopamine uptake are presented in Supplementary Figure S4). In all panels, the data are the mean + SEM of 3–10 independent experiments (performed using oocytes from different frogs), within which measurements were made from 10 oocytes per treatment. ^∗∗∗^ denotes a significant difference (*p* < 0.001) from the positive control (one-way ANOVA).

The inability of AmDATΔex3 to transport [^3^H]dopamine (Figure 2D) led us to investigate whether it may instead function to regulate the activity of the full-length transporter. The uptake of [^3^H]dopamine was measured in non-injected oocytes (negative control) and in oocytes expressing AmDAT (positive control) or AmDAT + AmDATΔex3. Two unrelated proteins – the *Plasmodium falciparum* nucleoside transporter (PfNT1) and the Emerald Green Fluorescent Protein (EmGFP) – were included as additional negative controls (neither protein affects the permeability of oocytes to dopamine; Figure 4 and Supplementary Figure S4). AmDAT was also expressed with PfNT1 or EmGFP to provide co-injection controls. We found that co-expression of AmDATΔex3 with AmDAT caused a 33 ± 8% decrease in the AmDAT-mediated transport of [^3^H]dopamine (mean ± SEM, *n* = 4; Figure 4 and Supplementary Figure S4). Moreover, this effect was sustained over a 3-day time-course. One possible explanation for this result would be competition between the co-injected cRNAs for the oocyte’s translational machinery. However, co-expression of AmDAT with PfNT1 or EmGFP had no effect on the dopamine transport activity of AmDAT (Figure 4). The co-expression of AmDAT with PfNT1 also had no effect on the PfNT1-mediated transport of hypoxanthine (PfNT1’s major substrate; Supplementary Figure S5).

#### Development of a Fluorescence-Based Assay to Quantitate EmGFP Levels

The expression of EmGFP in oocytes was measured using a novel fluorescence-based assay. We established that the assay was quantitative by measuring changes in the magnitude of the fluorescence signal when oocytes were injected with different amounts of EmGFP cRNA (ranging from 0 ng to 20 ng; Supplementary Figure S6). The level of fluorescence measured in non-injected oocytes (attributable to autofluorescence) was subtracted from each treatment and the fluorescence intensity was expressed as a percentage of the value obtained for the 10 ng treatment. A plot of the percent fluorescence intensity versus the amount of EmGFP cRNA injected produced a sigmoidal curve that was approximately linear between 0–10 ng of cRNA (Supplementary Figure S6B). We verified this result by conducting a western blot analysis of total protein extracts prepared from oocytes injected with 0–20 ng of EmGFP cRNA. A band corresponding to the predicted size of EmGFP was detected with an anti-GFP antibody and the band intensities were expressed as a percentage of the value obtained for the 10 ng treatment. The resulting plot of protein levels versus the amount of EmGFP cRNA injected revealed a sigmoidal relationship that was approximately linear between 0–10 ng of cRNA (Supplementary Figure S6A). Moreover, there was a strong correlation (*R*^2^ = 0.989) between fluorescence intensity and EmGFP protein levels in oocytes injected with 0–10 ng of EmGFP cRNA (Supplementary Figure S6C). Taken together, these findings confirmed that the fluorescence assay could be used to quantify the expression of EmGFP in oocytes injected with 0–10 ng of EmGFP cRNA.

#### AmDATΔex3 Does Not Inhibit the Translation of EmGFP

The application of the fluorescence assay revealed no differences in EmGFP levels between the following treatments on days 1–3 post-cRNA-injection: EmGFP-expressing oocytes (positive control), oocytes co-expressing EmGFP with AmDAT (co-injection control), oocytes co-expressing EmGFP with HA_EL__2_-AmDAT (co-injection control), and oocytes co-expressing EmGFP with AmDATΔex3 (*p* > 0.05; Supplementary Figure S7). The finding that EmGFP levels were unaffected by AmDATΔex3 suggests that the latter’s regulatory effects are not due to a general inhibition of the oocyte’s translational machinery. Moreover, the observation that the co-expression of AmDAT with PfNT1 failed to affect the expression of either membrane protein suggests that the regulatory effects of AmDATΔex3 cannot be attributed to competition with AmDAT for the ribosomes of the rough endoplasmic reticulum (ER).

#### AmDATΔex3 Downregulates the Expression of AmDAT

**FIGURE 5:**
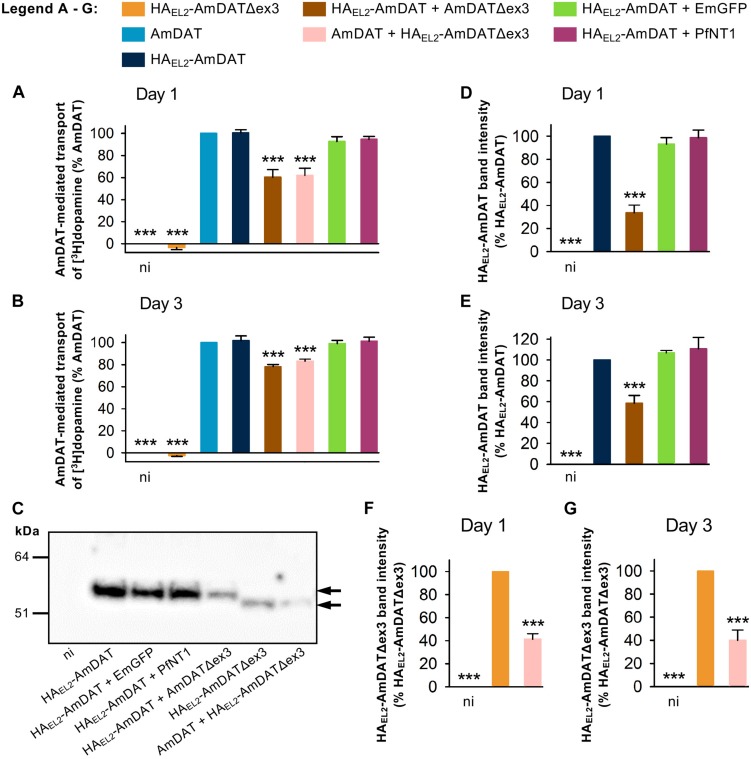
AmDATΔex3 downregulates AmDAT protein levels in *Xenopus* oocytes. The AmDAT-mediated transport of [^3^H]dopamine in oocytes expressing the HA-tagged forms of AmDAT or AmDATΔex3 was measured on **(A)** day 1 and **(B)** day 3 post-cRNA-injection. Non-injected oocytes (ni) were included as a negative control, EmGFP and PfNT1 were included as co-injection controls, and oocytes expressing AmDAT served as the positive control. Dopamine uptake was expressed as a percentage of that measured in the AmDAT-expressing oocytes. In both panels, the data are the mean + SEM of 3–6 independent experiments (performed using oocytes from different frogs), within which measurements were made from 10 oocytes per treatment. The capacity of HA_EL__2_-AmDAT for dopamine transport, and the ability of HA_EL__2_-AmDATΔex3 to downregulate AmDAT, did not differ significantly from that of its non-tagged counterpart (*p* > 0.05), consistent with the HA-tag exerting little or no effect upon the functions of these proteins. **(C)** Detection of the HA_EL__2_-AmDAT (∼55 kDa band indicated by black arrow) and HA_EL__2_-AmDATΔex3 (∼50 kDa band indicated by black arrow) proteins in oocyte membrane preparations. These western blot analyses were performed pairwise with the transport assays presented in panel **(A)**. The samples were separated on a SDS-polyacrylamide gel and probed with a mouse anti-HA antibody. The image is representative of >3 independent experiments (performed using oocytes from different frogs) and is shown in full in Supplementary Figure S8. Semi-quantification of the **(D,E)** HA_EL__2_-AmDAT and **(F,G)** HA_EL__2_-AmDATΔex3 proteins in the oocyte membrane preparations was performed on day 1 panels **(D,F)** and day 3 panels **(E,G)** post-cRNA-injection. Protein band intensities were expressed as a percentage of that measured for the relevant positive control (i.e., oocytes expressing either HA_EL__2_-AmDAT or HA_EL__2_-AmDATΔex3). The data in panels **(D–G)** are shown as the mean + SEM from 3–8 independent experiments. ^∗∗∗^ denotes a significant difference (*p* < 0.001) from the relevant positive control (one-way ANOVAs).

We undertook semi-quantitative western blot analyses to determine the effect of AmDATΔex3 on the protein levels of AmDAT. These experiments made use of both the HA-tagged and non-tagged versions of AmDAT and AmDATΔex3 (noting that the HA-tagged proteins exhibit the same [^3^H]dopamine transport activities and/or regulatory effects as their non-tagged counterparts; Figures 2D, 5A,B). We found that co-expression of HA_EL__2_-AmDAT with AmDATΔex3 caused a marked reduction in the level of HA_EL__2_-AmDAT protein relative to that measured in oocytes expressing only HA_EL__2_-AmDAT (the positive control) (Figure 5C and Supplementary Figure S8). By contrast, co-expression of HA_EL__2_-AmDAT with PfNT1 or EmGFP had no impact on HA_EL__2_-AmDAT protein levels. The down-regulatory effect of AmDATΔex3 was evident on both days 1 and 3 post-cRNA-injection (Figures 5C–E) and was not due to uneven sample loading and/or uneven transferral of proteins from the SDS-PAGE gel (Supplementary Figure S9). Moreover, the expression of AmDATΔex3 or HA_EL__2_-AmDATΔex3 did not affect the overall level of protein in the membrane preparations (Supplementary Figures S9A–C), which indicates that the splice variant does not significantly alter the levels of endogenous membrane proteins. Taken together, these observations reveal that the splice variant reduces the expression of AmDAT, that this decrease is evident at the protein level, and cannot be attributed to competition for ER ribosomes or a general inhibition of the oocyte’s translational machinery. It is also worth noting that there was a marked reduction in the level of HA_EL__2_-AmDATΔex3 protein in oocytes expressing AmDAT + HA_EL__2_-AmDATΔex3 relative to that measured in oocytes expressing only HA_EL__2_-AmDATΔex3 (Figures 5F,G). That is, the expression of both the full-length protein and its splice variant are significantly decreased in oocytes co-expressing AmDAT with AmDATΔex3.

### Expression of *amdat* and *amdat*Δ*ex3* in the Honey Bee

Having established that AmDAT is a dopamine transporter and that AmDATΔex3 can downregulate its expression in the oocyte system, we used qPCR to evaluate the expression of *amdat* and its splice variant in female castes and drones. These analyses confirmed our *in silico* findings that both the full-length transcript and *amdat*Δ*ex3* are expressed in adult brains, antennae, and queen ovaries. However, the *amdat*Δ*ex3* transcript level is generally very low relative to the level of *amdat* transcript (Supplementary Figure S10).

**FIGURE 6:**
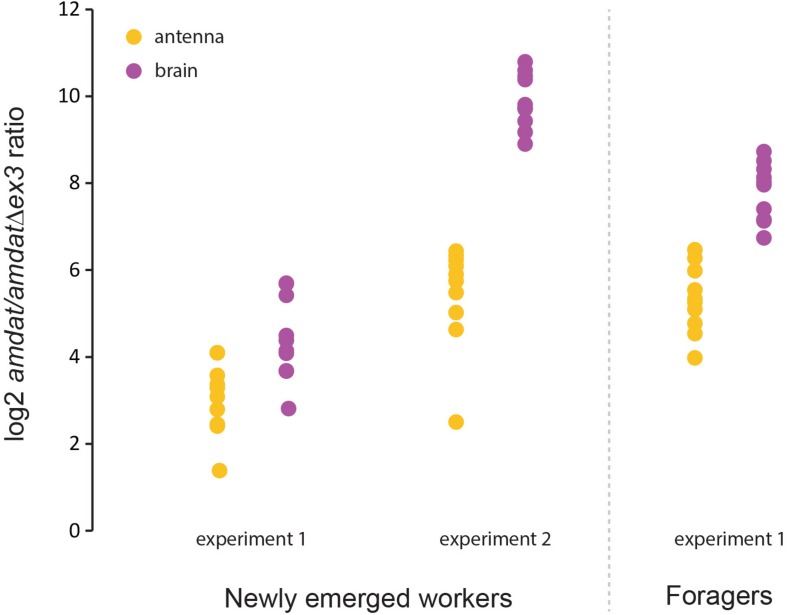
Interindividual variability in the expression of the *amdat* and *amdat*Δ*ex3* transcripts. RNAs extracted from single brains were used for qPCR evaluation. Ten individuals were analyzed in each experiment. Refer to the Supplementary Material for further details.

Given the high level of genetic diversity within the honey bee colony arising from the queen’s polyandry, as well as the expectation that AmDAT will be important for social behavior, we reasoned that gene expression in individual honey bees may be a more informative approach for evaluating the dynamics of the two *amdat* transcripts. Indeed, variability in gene expression between honey bee workers might be one way by which task-specialization occurs, including queen-worker communications. To assess *amdat*:*amdat*Δ*ex3* expression in individual bees (age-matched), we used brains extracted from newly emerged bees and older foragers. As shown in Figure 6, the levels of both transcripts were highly variable between the brains of individual worker bees. However, the relative expression of *amdat* versus *amdat*Δ*ex3* appears to be maintained at a similar level, with the full-length transcript always being the predominant form. Although we were unable to uncover a clear correlation between the level of *amdat*Δ*ex3* transcript and a specific situation in this study, the finding that this splice variant is always present at low frequencies in the brain and in some other tissues (Supplementary Table S3) indicates that it is likely to fulfill an important physiological role.

*In situ* hybridization with an *amdat* antisense probe produced a highly localized signal around a few clusters of neurons in the honey bee brain, thus confirming the expression of *amdat* in cells inferred to be involved in dopaminergic transmission on the basis of staining with anti-dopamine (Schäfer and Rehder, 1989; Schürmann et al., 1989) and with anti-tyrosine hydroxalase antibodies (Schäfer and Rehder, 1989; Schürmann et al., 1989; Tedjakumala et al., 2017; Supplementary Figure S11).

### Methylation of the *amdat* Locus Implies Epigenetic Regulation of the Transporter

Standard genome-wide profiling of DNA methylation in the honey bee brain suggested that there is very little methylation in the *amdat* locus, with just a single methyl-cytosine detected at the 3′ end (CpG#11 in Figure 1A). However, since only three out of the 14 SLC6 transporters encoded in the honey bee genome show signs of methylation, this observation suggested that epigenomic modifications may have been adopted to regulate the expression of these three transporters. This very low frequency of methyl-cytosine, which is consistent with a restricted spatial- or temporal-pattern of methylation, makes it difficult to draw conclusions about the methylation dynamics of *amdat* from genome-wide, low-coverage methylomic profiles. To overcome this problem, we used gene-focused, ultra-deep amplicon sequencing. This approach yields thousands of long-reads corresponding to the region of interest and allows visualization of the levels, as well as the patterns, of methylation in individual cell types within the honey bee brain (Becker et al., 2016; Wedd et al., 2016). The analysis presented here examined whole brains (nurses and foragers) as well as forager antennae. Four amplicons encompassing the following regions were generated from bisulfite-converted DNA: intron 2, intron 3, exon 3, and exons 10–11 (Figure 1A). The results are summarized in Supplementary Table S4. The sequencing coverage for the brains was high (72,000 to 129,000 reads per brain amplicons) and 20,000 reads for the antennae. In all situations a very low level of *amdat* methylation was detected; using a conservative conversion rate of 95%, the level of 5′-C-phosphate-G-3′ dinucleotide (CpG) methylation in the two introns and in exons 10–11 was ∼5–8% of all sequence reads in the whole brain and ∼10% in the antennae (Supplementary Table S4). No methylation was detected in exon 3. These findings suggest that the *amdat* locus is methylated in only a limited number of neurons, most likely those expressing *amdat.* Whilst this finding requires additional confirmation, the methylation of *amdat* in non-dopaminergic neurons would have yielded a much higher proportion of methylated CpGs that the levels measured here. This observation is in agreement with our previous work linking DNA methylation to active transcription (Wedd et al., 2016).

## Discussion

Previous studies have highlighted the importance of dopaminergic transmission in insect sociality, especially in a most critical aspect of social behavior – the queen-worker communication system that ensures the functional coherence of thousands of individuals in a colony (Beggs et al., 2007; Vergoz et al., 2007). The involvement of dopamine in the pheromonal control of worker bees is well documented, as is the correlation between changes in the concentration of dopamine and the progression of worker behaviors (Jarriault and Mercer, 2012). Here we have addressed a fundamental gap in our understanding of the honey bee dopaminergic system by identifying an *Apis mellifera* dopamine transporter as well as an unusual splice variant of this protein, and through conducting in-depth characterizations of their expression and functions.

When expressed in the *Xenopus* oocyte system, both the non-tagged and HA-tagged versions of full-length AmDAT cause a substantial increase in the uptake of dopamine (∼12-fold increase in dopamine transport relative to non-injected oocytes). This robust transport signal allows detailed investigations of the protein’s transport properties. We found that AmDAT exhibits a high specificity for dopamine and noradrenaline transport, that it translocates tyramine as a minor substrate, and does not appear to transport octopamine, L-Dopa, or serotonin. The finding that the concentration-dependence of dopamine transport via AmDAT is sigmoidal suggests that the transporter undergoes allosteric substrate activation. These transport properties of AmDAT in *Xenopus* oocytes, which are similar to those of the *Drosophila* dopamine transporter DmDAT (Porzgen et al., 2001), are consistent with its strong sequence homology to known dopamine transporters from other species. The crystal structure of DmDAT in complex with two antidepressants was recently determined (Penmatsa et al., 2015), and given the high level of sequence conservation between AmDAT and DmDAT, the structure of AmDAT is likely to be very similar (if not identical) to that of the *Drosophila* transporter.

We show that cocaine is a reversible inhibitor of AmDAT and that cocaine itself is not a substrate of the transporter. These results, which are consistent with the reported effects of cocaine on dopamine transporters (Lluch et al., 2005; Beuming et al., 2008; Sullivan et al., 2008), add further support to our recent identification of a link between cocaine and DNA methylation dynamics in the honey bee (Sovik et al., 2018). We showed that cocaine treatment interferes with memory processing independently of incentive salience by directly altering DNA methylation dynamics (Sovik et al., 2018) and thus the expression of methylated genes. Because *amdat* is methylated, both its epigenetic status and expression are expected to be significantly changed by cocaine, thereby leading to dopamine imbalance. In mammalian brains, many addictive drugs alter dopaminergic neurotransmission in the midbrain pathway by either enhancing the release or reducing the clearance of dopamine (Kuhar et al., 1991; Huang et al., 2009), and thereby increasing the incentive salience of a given stimuli (Berridge, 2007). Cocaine induces widespread changes in DNA methylation patterns in the mammalian brain (Nestler, 2014), which is of particular interest given that DNA methyltransferases (DNMTs) and demethylases are vital for memory formation in both mammals and honey bees (Barron et al., 2009; Lockett et al., 2010, 2012; Day and Sweatt, 2011; Wojciechowski et al., 2014). Because the impact of cocaine on behavior and neurochemical responses in bees and mammals are comparable (Barron et al., 2009; Sovik et al., 2018), this insect presents a valuable system by which to explore the basic interactions between drugs of abuse, epigenomic modifications, and animal behavior (Maleszka, 2016).

We find *amdat* to be predominantly expressed in the brain and antennae, but the presence of RNAseq reads in other tissue samples indicate that dopamine has roles outside the nervous system – including in the ovaries and glands. In comparison to the full-length mRNA, the expression of *amdat*Δ*ex3* is low and represents approximately 1% of the combined transcription. In age-matched individual bees, the expression of both transcripts in the brain is quite variable, which may be driven by genetic or epigenetic polymorphisms (Maleszka, 2016; Wedd and Maleszka, 2016; Wedd et al., 2016). Whether or not such variability in *amdat* and *amdat*Δ*ex3* expression is advantageous for social organization remains to be established, but it is conceivable that it could lead to differences in dopaminergic neurotransmission and thus differences in behavior. This is an area that has not been explored in honey bees or in other social insects, but some insights could be gained from mammalian research. For example, polymorphisms in the human DAT genes (e.g., HsDAT1) have been reported and associated with variations in susceptibilities to neurobiological disorders (Roman et al., 2004).

Our discovery and characterization of the AmDATΔex3 splice variant adds to the growing number of alternatively spliced transporters that have been shown to modulate the function of their full-length counterparts. For example, several naturally occurring splice variants of mammalian transporters have been reported to exert a dominant-negative effect on their full-length isoforms (Kitayama et al., 1999; Vallejo-Illarramendi et al., 2005; Gebhardt et al., 2010; Sogawa et al., 2010; De Bellis et al., 2014). Like AmDATΔex3, these alternatively spliced transcripts are missing one or more exons from their respective full-length sequences and are hence predicted to encode proteins with grossly deformed topologies. Moreover, thus far the majority of these splice variants are isoforms of mammalian neurotransmitter transporters, including the noradrenaline transporter (NET), DAT, and excitatory amino acid transporters 1 and 2 (EAAT1 and EAAT2, respectively). These studies have proposed a regulatory mechanism involving the formation of unstable heteromeric complexes, formed by the splice variant and full-length proteins, that are retained in the ER and targeted for degradation (Gebhardt et al., 2010; Sogawa et al., 2010; De Bellis et al., 2014). The expression of AmDATΔex3 may likewise cause AmDAT to be retained in the ER and targeted for degradation, but we cannot rule out the possibility that *amdat*Δ*ex3* instead acts by inducing the degradation of the full-length *amdat* transcript.

The presence of *amdat*Δ*ex3* transcripts in virtually all honey bee RNAseq libraries (albeit at low levels) suggests it has a physiological role. Although the mechanism underpinning the regulatory activity of AmDATΔex3 remains unresolved, it is possible that its activity is not limited to the modulation of full-length AmDAT. For example, the splice variant of rat NET was found to downregulate the expression of several rat neurotransmitter transporters – including DAT, other monoamine transporters, and the GABA transporter type 1 – but appeared to have no effect on EAAT1 (also known as GLAST) (Kitayama et al., 1999). Given the sequence similarities between the NET and DAT proteins, it is possible that the physiological role of AmDATΔex3 is to fine-tune the expression of AmDAT, and potentially other neurotransmitter transporters, via a mechanism that bypasses the more elaborate and expensive process of *de novo* transcription and translation of distinct regulatory proteins. This may enable more rapid cellular responses to developmental and/or environmental cues. The benefits of this mechanism for social interaction, which are often based on subtle threshold responses (Johnson, 2003; Jeanson and Weidenmuller, 2014; Wedd et al., 2017), could be plentiful. Hence, our demonstration of AmDATΔex3’s ability to downregulate the expression of AmDAT *in vitro* is a significant result that merits further investigation.

Our epigenetic analyses show that the *amdat* locus is modified by the methylation of cytosines in two introns and one exon (altogether a total of ten CpGs). Only two of the remaining 13 SLC6 transporter genes in the honey bee genome show signs of methylation (Supplementary Table S2). Using the ultra-deep amplicon sequencing approach, we found that the ten CpGs are methylated at ∼5–10% in whole-brain or antennae extracts. This result suggests that *amdat* is methylated in only a small proportion of cells. One possibility is that these cells correspond to dopaminergic neurons that consist of four small clusters in the honey bee brain [(Schäfer and Rehder, 1989; Schürmann et al., 1989; Tedjakumala et al., 2017) and (Supplementary Figure S11)]. Similarly, only the cells involved in dopaminergic communication in the antennae would carry methylated copies of *amdat*. These findings provide indirect support for the view that DNA methylation in the honey bee is associated with active transcription rather than repression (Kucharski et al., 2016; Wedd and Maleszka, 2016; Wedd et al., 2016; Welsh et al., 2017). It is worth noting that in the honey bee genome-wide methylomic datasets that were generated at low depth, only a single methylated CpG#11 is documented in the *amdat* locus (Lyko et al., 2010). Hence, an important insight gained from this study is that the epigenomic architecture of the dopaminergic (and similar) systems in the honey bee should be studied at the level of specific cell groups, rather than whole-brain extracts, when applying a low-depth sequencing approach. Whilst methylomic variations in the promoter region of the human DAT1 gene (SLC6A3) have been reported to affect DAT expression, these findings are of limited relevance to the honey bee because it is the gene bodies – not the promoters – that are methylated in insect genomes. Moreover, promoter methylation in mammals is associated with transcription repression, rather than its activation (Ziller et al., 2013).

Given the small number of clustered dopaminergic neurons in the honey bee brain, the whole-brain transcriptome or epigenome profiles are poor indicators of the biological relevance of AmDAT. This important aspect of brain research in honey bees, and other organisms, was emphasized in the context of dopamine receptors whose age-related expression in worker brains was found to differ depending on the functionally diverse groups of cells within the mushroom body neuropil (McQuillan et al., 2012). These and other findings (Maleszka, 2016) bring into focus the need to move beyond conventional analyses by developing cell-type-specific approaches at high depth to uncover the conditions affecting differential gene expression or methylation in defined neuronal populations, and how these dynamic processes affect brain circuitry and behavior.

Our study has generated important insights into the transciptome-epigenome-protein-level control of an important transporter that has been implicated in social behavior. Although many features of AmDAT are conserved, we have uncovered novel properties that distinguish the honey bee protein from its close homologs in insects and other organisms. These include the methylation of *amdat*, which is not possible in *Drosophila* or in the many other insect and invertebrate species that lack all (or some) of the components of epigenetic machinery (Lyko and Maleszka, 2011; Wedd and Maleszka, 2016). Importantly, our discovery of a non-transporting variant capable of reducing the expression of full-length AmDAT implies an additional level of regulation that could impact other neuromodulatory circuits interacting with dopaminergic transmission. When combined with a significant level of inter-individual variability of *amdat* expression, the multiple levels of control could contribute to subtle behavioral adjustments that are an important feature of eusociality (Maleszka et al., 2014; Maleszka, 2016, 2018). In a broader context this work reinforces the utility of the honey bee in modeling the extent to which intricate cellular networks can be used to reshape phenotypic outcomes. Our findings encourage new avenues of research that seek to understand how social interactions are being driven by lineage-specific tuning of otherwise conserved signaling pathways.

## Data Availability Statement

All datasets generated for this study are included in the article/Supplementary Material. Additionally, ultra-deep sequencing reads will be provided upon request.

## Ethics Statement

Ethical approval of the work performed with the *Xenopus laevis* frogs was obtained from the Australian National University Animal Experimentation Ethics Committee (Animal Ethics Protocol Number A2011/32 and A2016/12) in accordance with the Australian Code of Practice for the Care and Use of Animals for Scientific Purposes.

## Author Contributions

VZ, RK, CL, and SR performed the experiments and acquired the data. RM conceived the original project. REM, RM, VZ, RK, CL, SR, and SB contributed to the design of work as well as the analysis and interpretation of the data. RM, REM, and SB supervised the study. VZ, SR, and RK contributed to drafting the manuscript. REM and RM wrote the manuscript.

## Conflict of Interest

The authors declare that the research was conducted in the absence of any commercial or financial relationships that could be construed as a potential conflict of interest.
