# Supplementary material for: Characterization of a Dopamine Transporter and Its Splice Variant Reveals Novel Features of Dopaminergic Regulation in the Honey Bee
Source: Front Physiol. 2019 Nov 1;10:1375. doi: 10.3389/fphys.2019.01375 (PMC6838227; doi:10.3389/fphys.2019.01375)
Supplement: Supplementary file 1 [file Data_Sheet_1.pdf]

Figure S1

Cloning strategy of *amdat* and *amdatΔex3in*
See a detailed description in Supplementary Data and Methods (page 3)

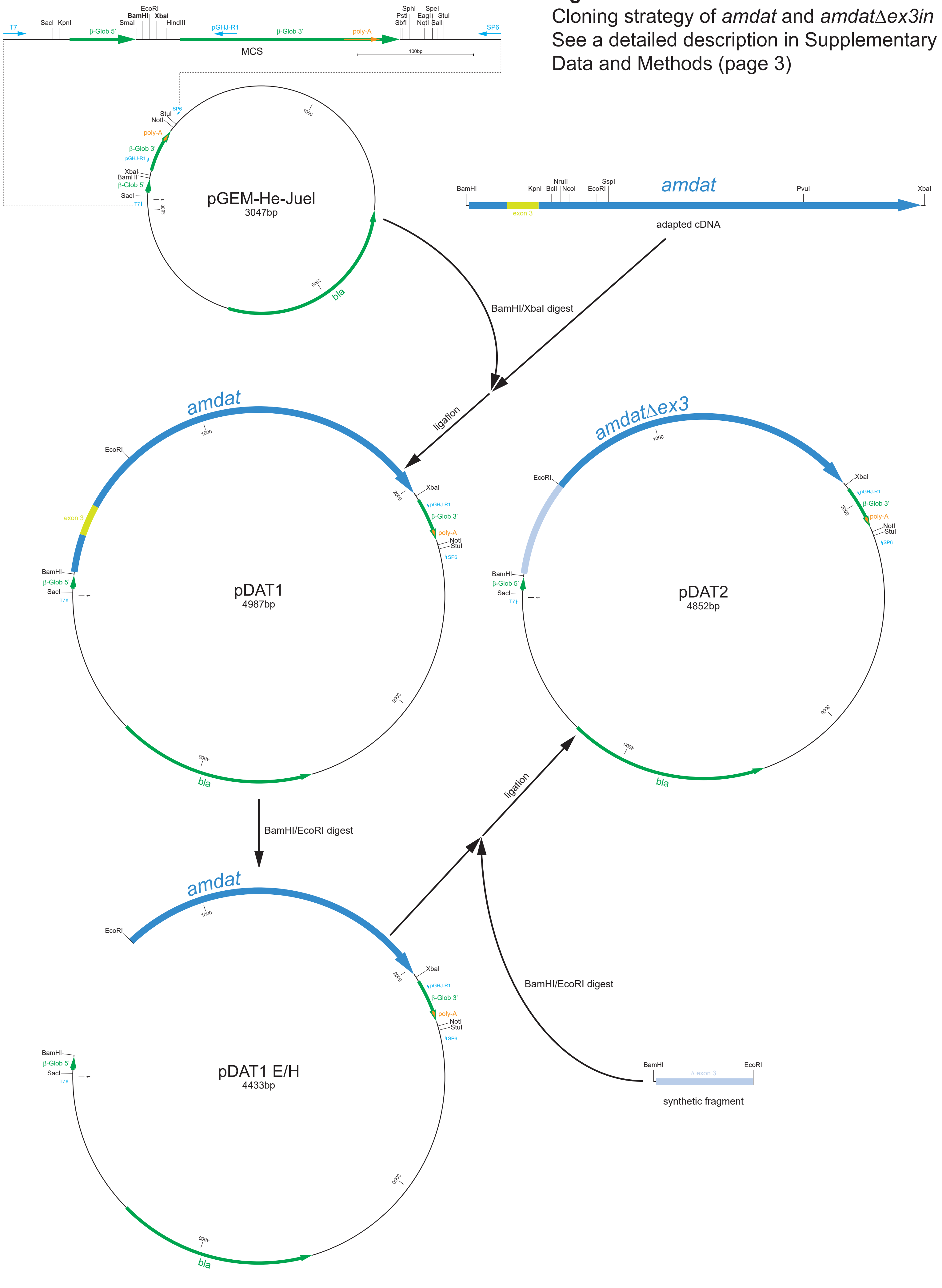

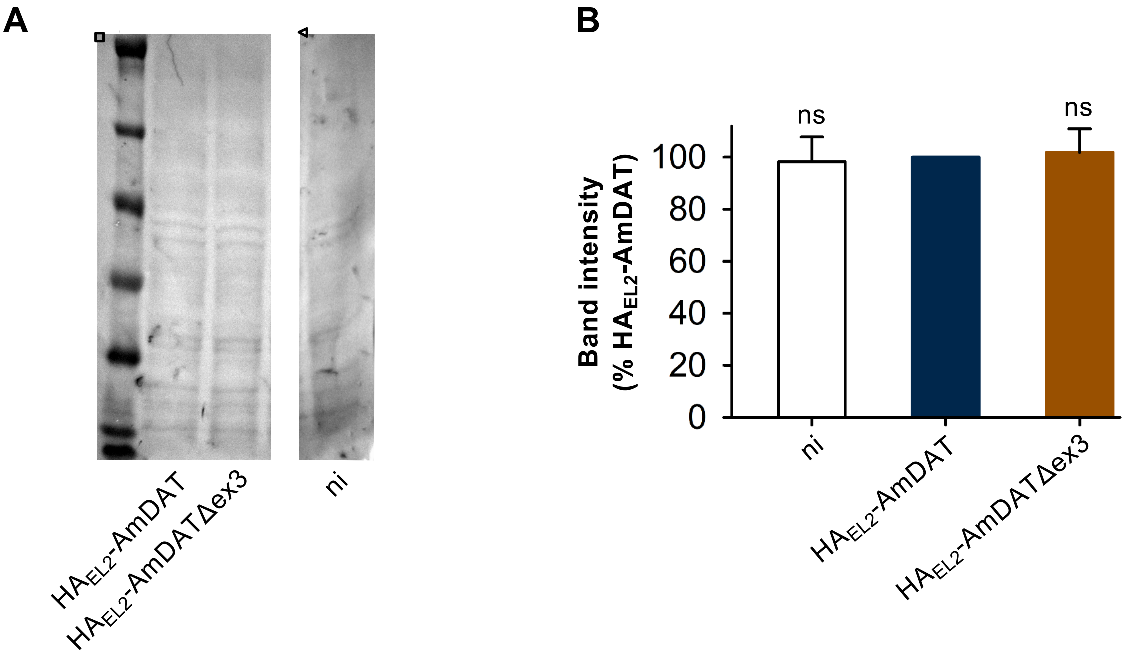

Supplementary Figure 2. Total protein levels in the western blot analyses of oocytes expressing HA_{EL2}-AmDAT or HA_{EL2}-AmDATΔex3. **(A)** A representative image depicting the total protein present in samples prepared from oocytes expressing HA_{EL2}-AmDAT or HA_{EL2}-AmDATΔex3. Non-injected oocytes (ni) were included as a negative control. Preparations of oocyte membrane proteins were separated on a SDS-polyacrylamide gel and transferred to a nitrocellulose membrane. The proteins were visualised with the MemCode^{TM} reversible protein stain kit and the image is representative of $n = 6$. **(B)** The amount of protein present in each lane was semi-quantified using Image Studio Lite and the resulting values were expressed as a percentage of that measured for the HA_{EL2}-AmDAT lane. Total protein levels are shown as the mean + SEM from 6 independent experiments performed on day 3 post-cRNA-injection. ns, no significant difference ($p > 0.05$) from the HA_{EL2}-AmDAT lane (one-way ANOVAs).

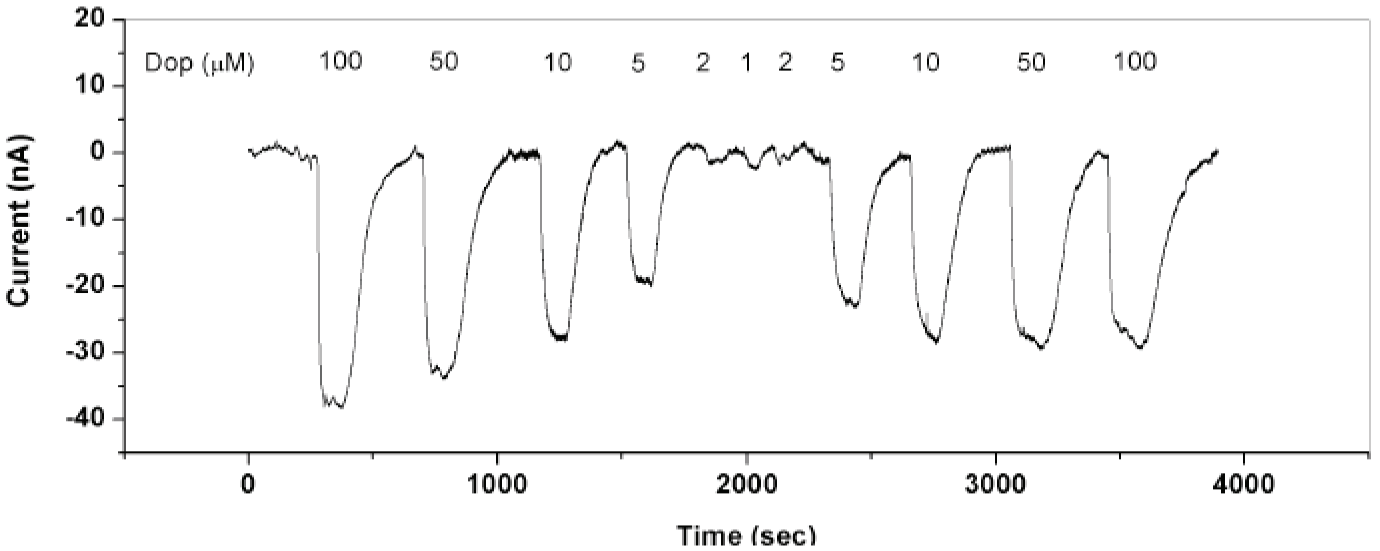

Figure S3A. Response of AmDAT to different concentrations of dopamine
Oocytes were injected with AmDAT cRNA and incubated for 3 days. Subsequently, oocytes were held at a membrane potential of -50mV and superfused with ND96 (pH 7.4) alone or ND96 (pH 7.4) containing dopamine at final concentrations indicated above each peak in μM. Plot represents the responses of a single oocyte. Dop: dopamine

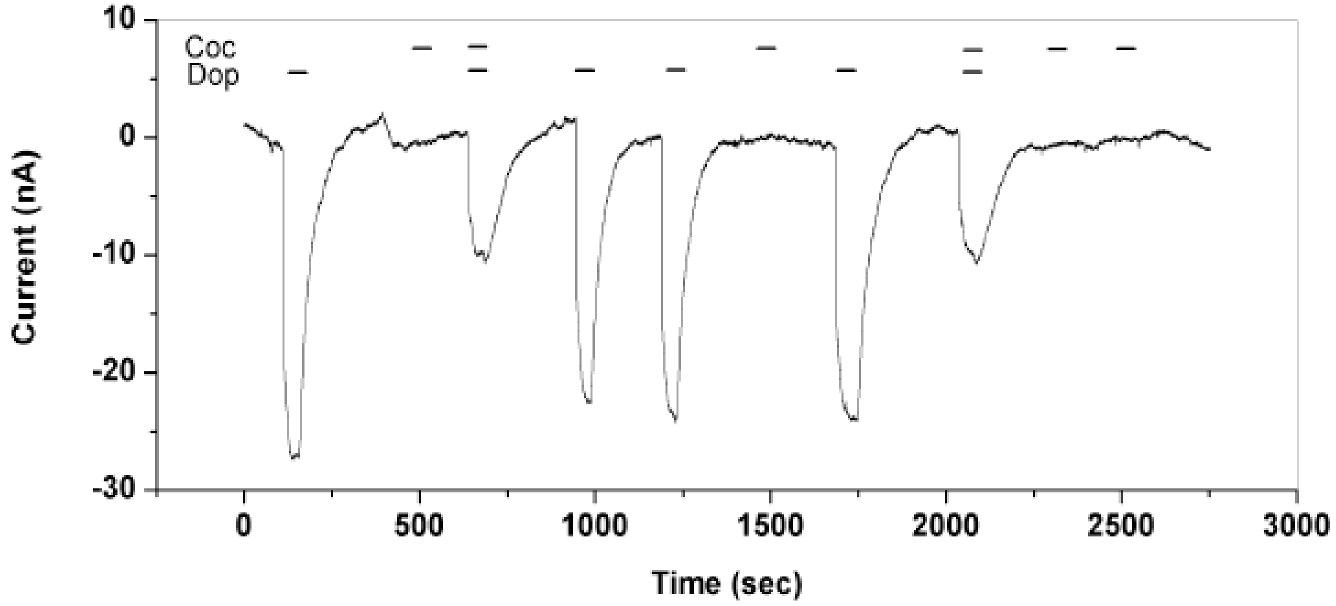

Figure S3B. Inhibition of dopamine transport by AmDAT by the presence of cocaine.
The figure presents an example of the inability of cocaine to produce a current in AmDAT expressing oocytes, supported by a significantly smaller normalised current
(Mean 3.4 S.E.M. 1.7 $p<0.001$). Currents induced with a dopamine/cocaine mix were significantly smaller than currents induced by dopamine alone
(Mean 61.6 S.E.M. 4.9 $p < 0.001$) (Figure 3), indicating the inhibition of AmDAT-mediated dopamine transport by cocaine. Currents could still be induced with dopamine after superfusion with cocaine or a dopamine/cocaine mix, showing that cocaine binds to AmDAT in a reversible manner.

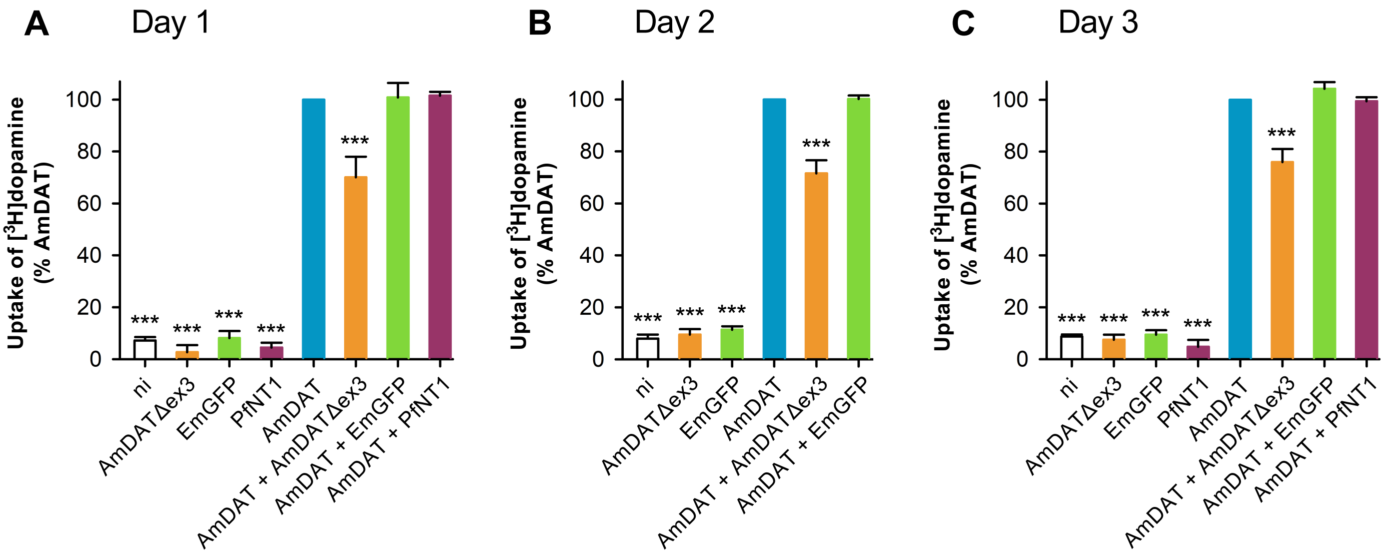

Supplementary Figure 4. The uptake of dopamine into oocytes co-expressing AmDAT and AmDATΔex3. Measurements of [^{3}H]dopamine transport were performed on **(A)** day 1, **(B)** day 2, and **(C)** day 3 post-cRNA injection. Non-injected oocytes (ni) were included as a negative control, EmGFP and PfNT1 were included as co-injection controls, and oocytes expressing AmDAT served as the positive control. Dopamine uptake was expressed as a percentage of that measured in AmDAT-expressing oocytes. The rates of dopamine uptake (pmol per oocyte/h) in non-injected oocytes and oocytes expressing AmDAT were 0.33 ± 0.07 and 4.92 ± 1.11 (day 1), 0.34 ± 0.02 and 3.86 ± 1.63 (day 2), and 0.65 ± 0.09 and 7.07 ± 1.37 (day 3), respectively. The data are the mean + SEM of 3–10 independent experiments (performed using oocytes from different frogs), within which measurements were made from 10 oocytes per treatment. *** denotes a significant difference ($p < 0.001$) from the positive control (one-way ANOVAs).

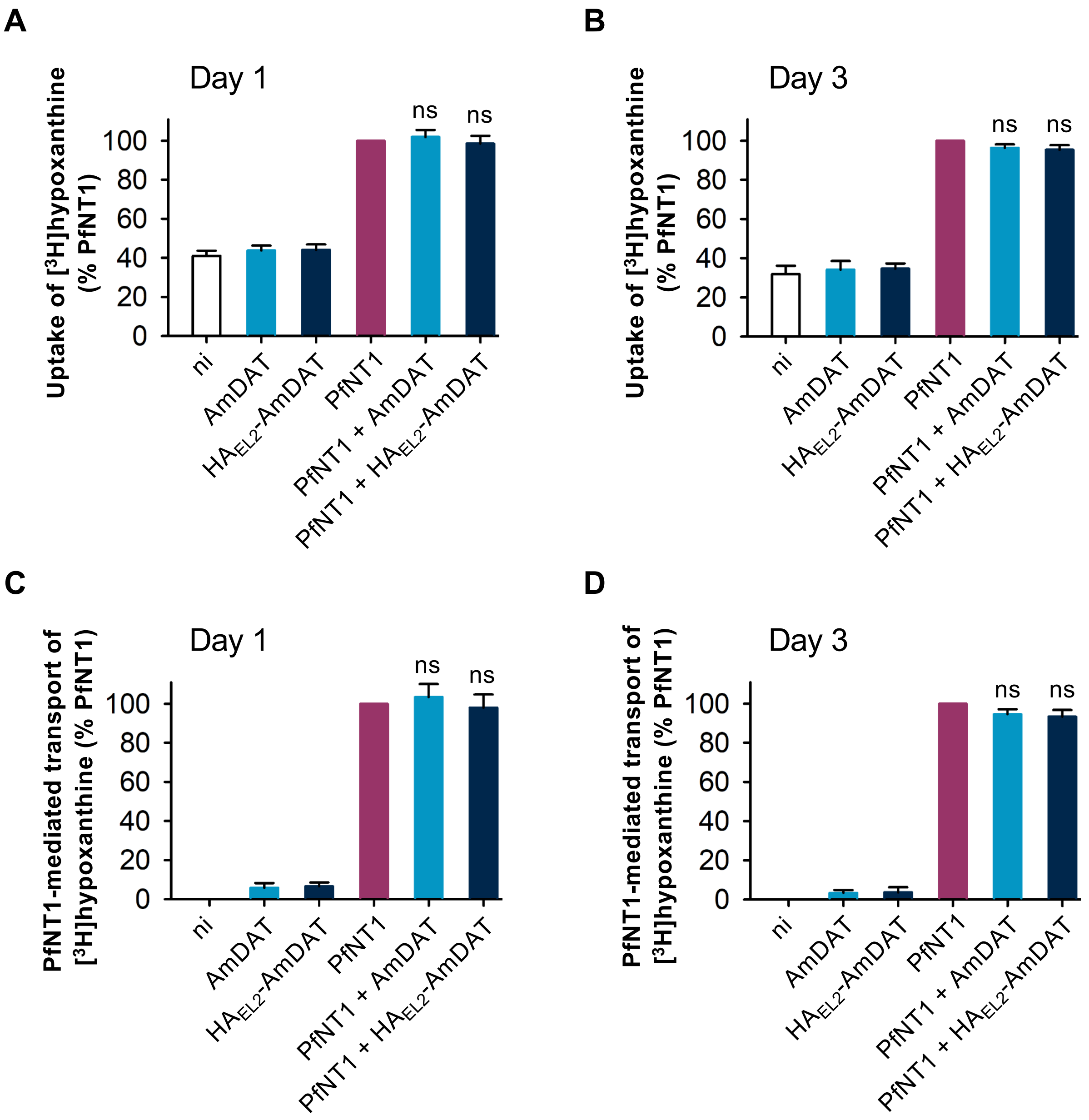

Supplementary Figure 5. The uptake of hypoxanthine into *Xenopus* oocytes co-expressing PfNT1 with AmDAT or HA_{EL2}-AmDAT. **(A,B)** Measurements of [^{3}H]hypoxanthine transport were performed on days 1 and 3 post-cRNA-injection. Non-injected oocytes (ni) were included as a negative control, and oocytes expressing PfNT1 served as the positive control. Hypoxanthine uptake was expressed as a percentage of that measured in PfNT1-expressing oocytes. The rates of hypoxanthine uptake (pmol per oocyte/h) in non-injected oocytes and oocytes expressing PfNT1 were 1.01 ± 0.17 and 2.45 ± 0.34 (day 1), and 1.43 ± 0.40 and 4.30 ± 0.60 (day 3), respectively. **(C,D)** Using the data shown in panels **(A,B)**, the component of [^{3}H]hypoxanthine transport attributable to PfNT1 was calculated by subtracting the background level of accumulation (i.e. the uptake measured in non-injected oocytes) from that measured for each of the oocyte types. In both panels, the data are the mean + SEM of 3–4 independent experiments (performed using oocytes from different frogs), within which measurements were made from 10 oocytes per treatment. ns, no significant difference ($p > 0.05$) in hypoxanthine accumulation relative to the PfNT1-expressing oocytes (one-way ANOVAs).

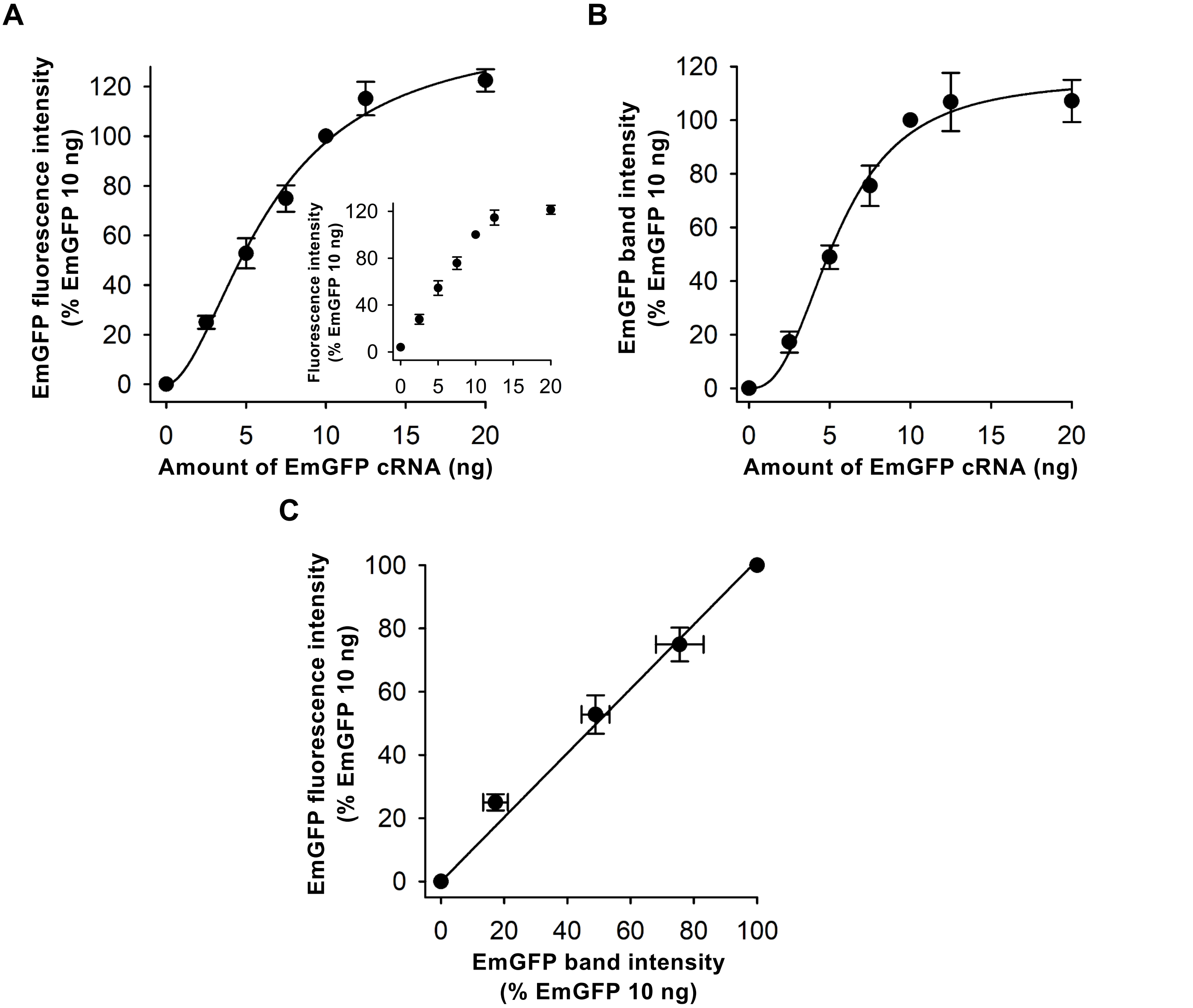

Supplementary Figure 6. Semi-quantification of EmGFP expression in *Xenopus* oocytes. **(A)** Oocytes injected with 0–20 ng of EmGFP cRNA were lysed and the total fluorescence intensity (inset plot) measured using excitation and emission wavelengths of 487 nm and 509 nm, respectively. The component of fluorescence attributable to EmGFP (main plot) was calculated by subtracting the background level of fluorescence (i.e. the autofluorescence detected in lysates of non-injected oocytes) from that measured in lysates of oocytes expressing EmGFP. In both plots of **(A)**, fluorescence was expressed as a percentage of that measured for the 10 ng treatment. The resulting plots revealed a sigmoidal relationship between the amount of EmGFP cRNA injected and the fluorescence intensity measured in the oocyte lysates (four-parameter logistic equation; $R^2 = 0.992$). **(B)** In experiments performed pairwise with those presented in panel **(A)**, the correlation between the amount of EmGFP cRNA injected and the resulting level of EmGFP expression was examined with a western blot protocol (Marchetti et al., 2015). Oocytes were injected with 0–20 ng of cRNA encoding EmGFP and whole oocyte extracts were prepared on day 3 post-cRNA-injection for analysis by western blot (using an anti-GFP antibody). The intensities of the resulting EmGFP bands were expressed as a percentage of that measured for the 10 ng treatment. Plotting these values against the amount of EmGFP cRNA injected revealed a sigmoidal relationship between the amount of EmGFP cRNA injected and the intensity of the EmGFP band (four-parameter logistic equation; $R^2 = 0.992$). The relationship remained approximately linear when ≤ 10 ng of EmGFP cRNA was injected into the oocytes. The relationship remained approximately linear when ≤ 10 ng of EmGFP cRNA was injected into the oocytes. **(C)** Combining plots **(A,B)** over the 0–10 ng treatments confirmed a positive correlation ($R^2 = 0.989$) between EmGFP band intensity and EmGFP fluorescence intensity. Taken together, these datasets indicated that measurements of EmGFP fluorescence intensity can be used to quantify EmGFP protein expression in the lysates of oocytes injected with ≤ 10 ng of EmGFP cRNA. In all panels, the data are the mean ± SEM of 3–5 independent experiments (performed using oocytes from different frogs) and were undertaken on day 3 post-cRNA-injection.

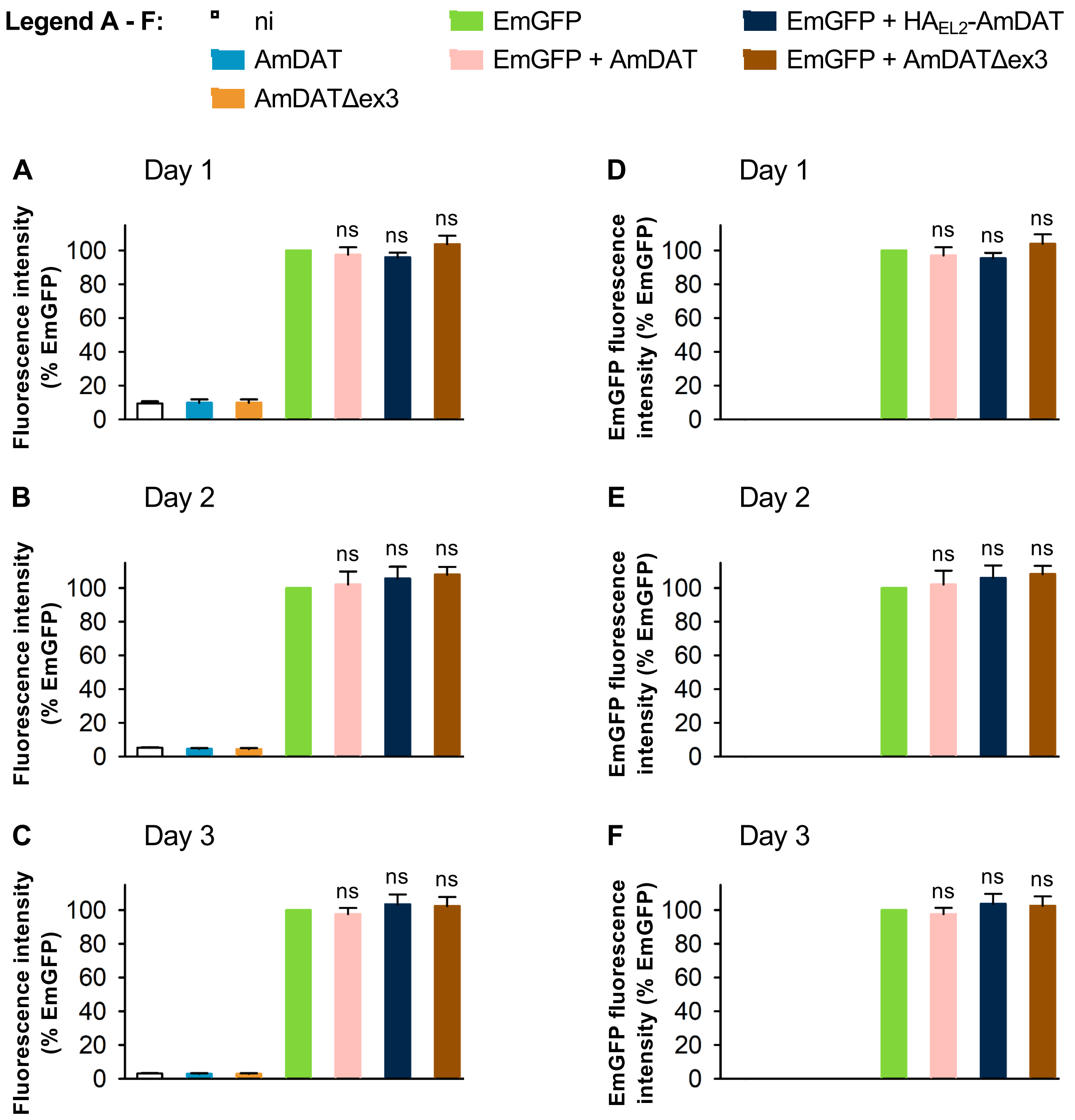

Supplementary Figure 7. Co-expression of EmGFP with AmDAT or AmDATΔex3 in *Xenopus* oocytes. Lysates of oocytes co-expressing EmGFP with AmDAT, HA$_{EL2}$-AmDAT, or AmDATΔex3 were prepared on days 1–3 post-cRNA-injection. The negative controls included non-injected oocytes as well as oocytes expressing AmDAT or AmDATΔex3, and oocytes expressing EmGFP served as the positive control. **(A–C)** Fluorescence intensity was measured using excitation and emission wavelengths of 487 nm and 509 nm, respectively, and expressed as a percentage of that measured in the EmGFP lysates. **(D–F)** The component of fluorescence attributable to EmGFP was calculated by subtracting the background level of fluorescence (i.e. the autofluorescence detected in lysates of non-injected oocytes) from that measured for each of the oocyte types. In all panels, the data are the mean + SEM of 3–7 independent experiments (performed using oocytes from different frogs), within which measurements were made from 10 oocytes per treatment. ns, no significant difference ($p > 0.05$) from the positive control (one-way ANOVAs).

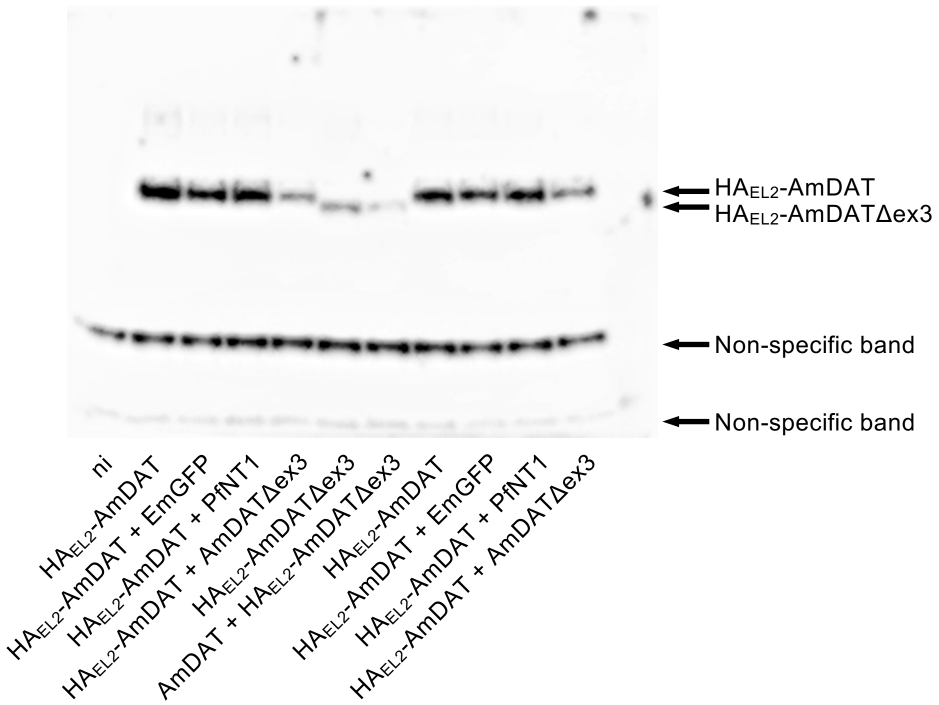

Supplementary Figure 8. Detection of the HA_{EL2}-AmDAT and HA_{EL2}-AmDATΔex3 proteins in oocyte membrane preparations. A cropped version of this figure is presented in Figure 5C. The samples were separated on a SDS-polyacrylamide gel and probed with a mouse anti-HA antibody. Bands corresponding to the predicted sizes of AmDAT and AmDATΔex3 were detected in samples prepared from oocytes expressing HA_{EL2}-AmDAT or HA_{EL2}-AmDATΔex3, respectively, and were absent from the samples prepared from non-injected (ni) oocytes. Two protein bands appeared in the lower half of the western blot image; the presence of these bands in all oocyte samples indicates they are endogenous oocyte proteins that reacted non-specifically with the primary or secondary antibody. The image is representative of > 3 independent experiments (performed using oocytes from different frogs).

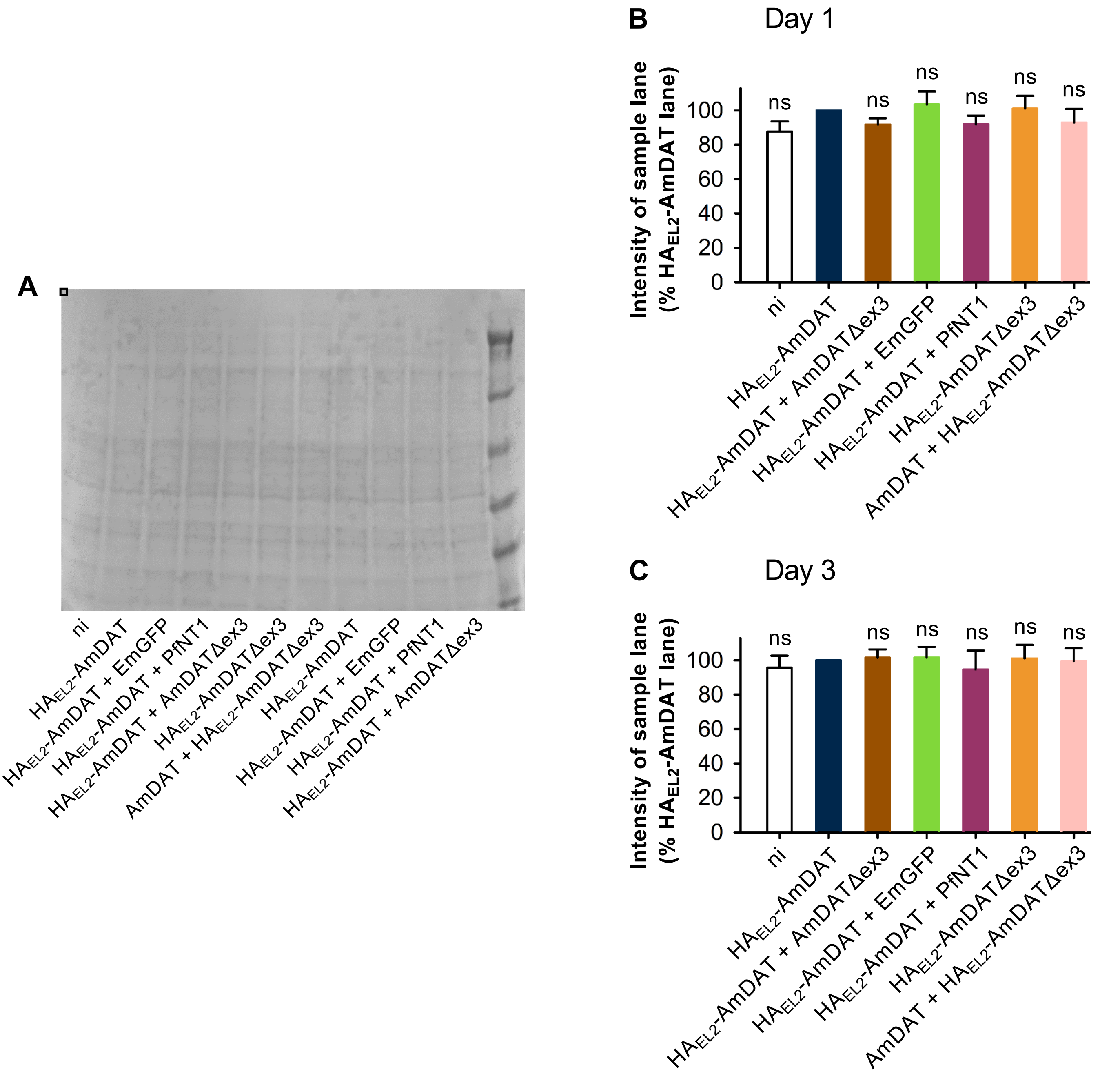

Supplementary Figure 9. Total protein levels in the western blot analyses of oocytes co-expressing AmDAT and AmDATΔex3. **(A)** A representative image depicting the total protein present in samples prepared from oocytes co-expressing HA_{EL2}-AmDAT with AmDATΔex3, EmGFP, or PfNT1, and from oocytes co-expressing AmDAT and HA_{EL2}-AmDATΔex3. Non-injected oocytes (ni) were included as a negative control and oocytes expressing HA_{EL2}-AmDAT served as the positive control. Preparations of oocyte membrane proteins were separated on a SDS-polyacrylamide gel and transferred to a nitrocellulose membrane. The proteins were visualised with the MemCode^{TM} reversible protein stain kit and the image is representative of n = 8. **(B,C)** The amount of protein present in each lane was semi-quantified using Image Studio Lite and the resulting values were expressed as a percentage of that measured for the HA_{EL2}-AmDAT lane. Total protein levels are shown as the mean + SEM from 3–8 independent experiments performed on days 1 and 3 post-cRNA-injection. ns, no significant difference (p > 0.05) from the positive control (one-way ANOVAs).

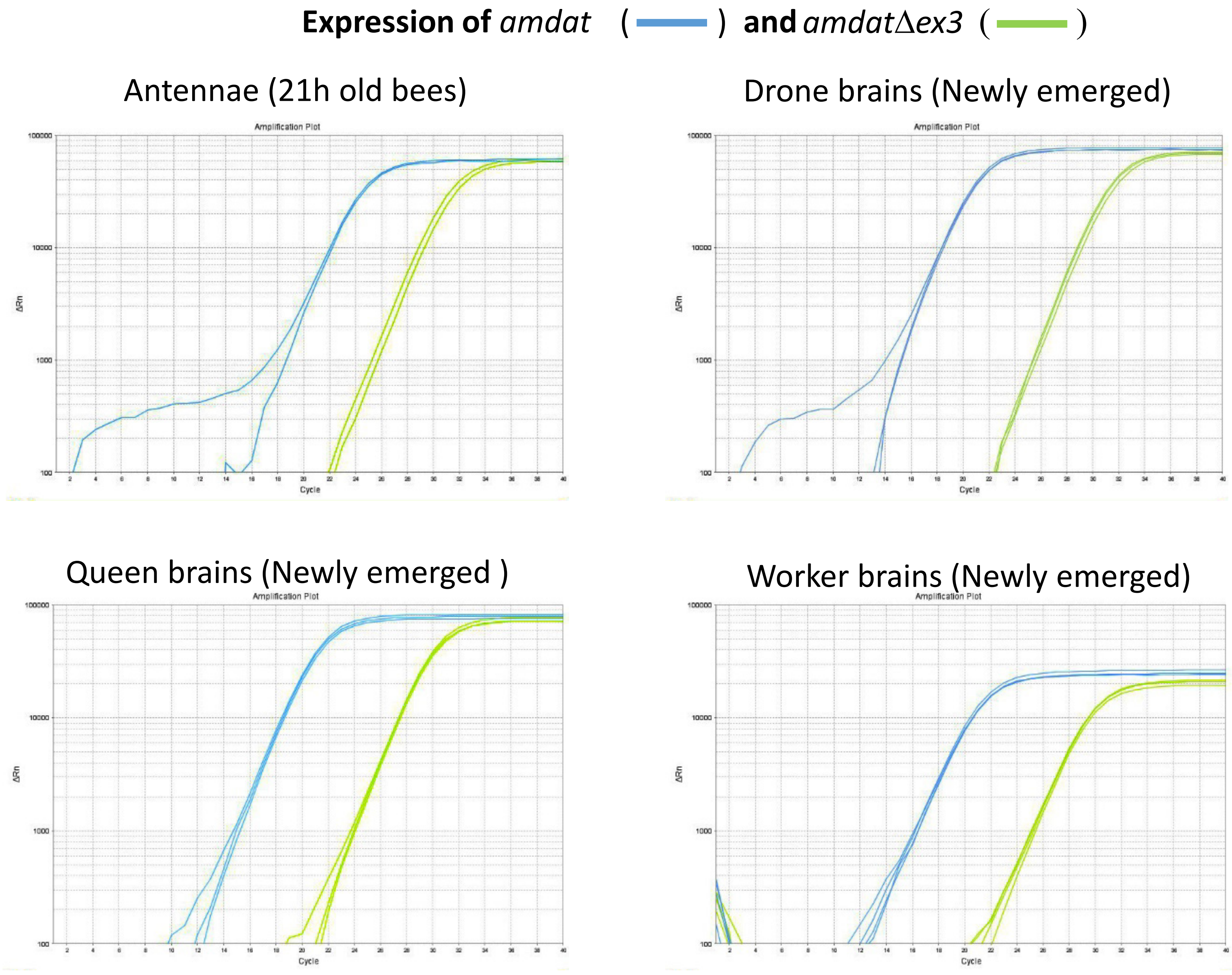

Figure S10. qPCR analysis of *amdat and amdatΔex3* expression in various situations. More details in Supplementary Materials.

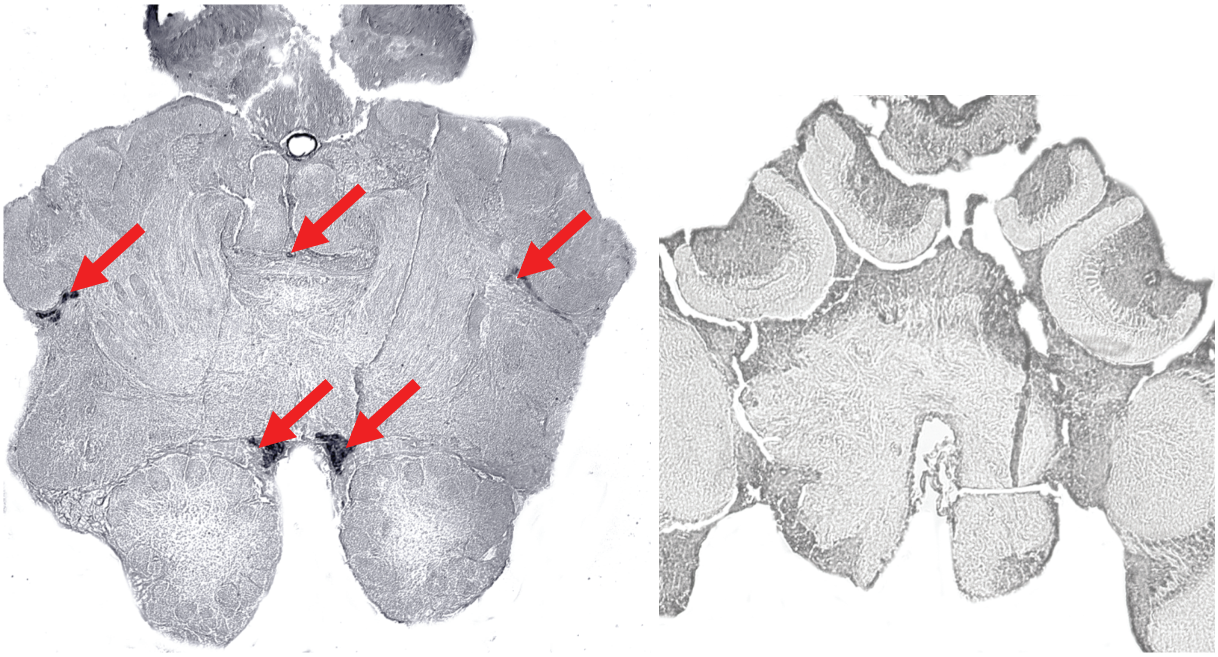

Figure S11. In situ hybridization showing the localization of the amdat transcript in the honey bee brain (adult worker). Dopamine interneurones somata clusters are indicated by arrows. See Supplementary Material for more details.

Table S1. Polymorphisms in cloned GB15426 fragment detected by sequencing

SNP#	Nucleotide change	Codon position	Amino acid	AA change?
1	c510T>C	3	P	No
2	c537G>T	3	R	No
3	c804C>A	3	G	No
4	c807T>C	3	V	No
5	c810A>G	3	T	No
6	c940T>C	1	L	No
7	c974T>C	3	N	No
8	c978C>T	3	V	No
9	c1257T>C	3	S	No
10	c1398T>C	3	Y	No
11	c1431T>C	3	I	No
12	c1596T>C	3	Y	No

Table S2. List of *A. mellifera* SLC6 genes annotated by this investigation

Id v.2.0	Id v.4.5	mRNA	Protein	Splicing	mCpGs
GB30094[1]	GB51834	1,872	623	No	No
GB30093[1]	GB51833	1,854	617	No	No
GB13527[1]	GB43519	1,746	581	No	No
GB15100[1]	GB51844	1,956	651	No	No
GB18122	GB44121	2,103	700	Yes	No
**GB15426**	**GB40867**	**1,932**	**643**	**Yes**	**Yes**
GB16752	GB51198	1,941	646	No	Yes
GB18205	GB46300	2,667	888	No	No
GB19183	GB49396	1,926	641	No	No
GB19372	GB54918	1,572	523	No	No
GB14492	GB51190	2,010	669	No	No
GB14272	GB50262	1,926	641	Yes	No
GB17568	GB44295	2,193	730	No	No
GB18859	GB47438	2,457	818	Yes	Yes

Genes are listed using official ‘GBXXXXX’ nomenclature in genome assembly 2.0 and 4.5. mRNA length is given in nucleotides. Protein length is given in amino acids. mRNA length and protein length refer to the longest complete gene model, as used in phylogenetic tree construction. [1] Genes found in tandem within the *A. mellifera* genome. mCpGs- methylated CpGs found in these loci.

Table S3. Identification of the truncated splice variant of *amdat*

The truncated AmDAT isoform was first discovered by analysing 454 RNA-seq reads mapped to *Apis mellifera* v.2.0 assembly with BLAT aligner (mapping by S. Foret; BLAT reference: Kent WJ. BLAT - the BLAST-like alignment tool. Genome Res. 2002 Apr;12(4):656-64). One read from pooled brain and ovary mRNA sample (ID: SRR063948.233094) showed an unusual splicing pattern.. The isoform was later detected in numerous honeybee RNA-seq datasets as shown below by BLAST-ing a 60 nucleotide, isoform-specific probe against NCBI SRA database: https://www.ncbi.nlm.nih.gov/sra/

**NCBI Id**	**Source of tissue**
SRR1239305	Antenna polyA RNA of *Apis mellifera* Nurse C1
SRR3033264	Eviscerated abdomens with attached fat bodies and ovaries
SRR071809	Nurse brain 3
SRR071819	Forager brain 3
SRR567649	Forager Brain
SRR1254948	Hypopharyngeal gland polyA RNA of *Apis mellifera* Forager (Colony 3)
SRR1255010	Mandibular Gland polyA RNA of *Apis mellifera* Forager (Colony 2)
SRR1255154	Nasonov gland polyA RNA of *Apis mellifera* Nurse (Colony 1)
SRR806711	sting glands from nurses from colony 2
SRR806709	forager sting glands from colony 2
SRR802538	forager sting glands from colony 1
SRR2034253	S. alvi + L. passim inoculated; Incubator maintained

Table S4. Methylation of the *amdat* locus

Intron 2							Intron 3		
CpG#	1	7	9	13	17	Coverage	23	27	Coverage
Brain	5.3%	6.2%	6.2%	5.8%	5.7%	129,633	6.9%	6.1%	73,863

Exons 10-11				
CpG#	4	7	11	Coverage
Brain 1	7.8%	5.4%	6.7%	125,022
Brain 2	8.4%	-	7.2%	72,678
Antennae	-	-	10.4%	20,000

Significantly methylated CpGs in the *amdat* locus. No methylation was detected in the amplicon spanning exon 3. Brain-1: newly emerged workers, Brain-2: 5-day-old workers. Antennae were from 2-3 weeks old foraging bees. The observed methylation of CpG #7 in exon 10-11 in sample Brain-2 and CpGs #4 and #7 in antennae didn't meet the statistical criteria to be considered significant. ($p<0.05$ after FDR correction, binomial test with conservative bisulfite conversion efficiency value of 95% used). See Figure 1 and Supplementary Materials for more information regarding the amplicon design and sequencing.

Supplementary Material

1 Supplementary Data and Methods

DNA methylation analysis by deep amplicon sequencing

DNA bisulfite conversion and amplicon preparation

1 µg of genomic DNA was bisulfite converted using the QIAGEN Epitect® Bisulfite Kit, as per the manufacturer's protocol. The converted DNA was amplified via a nested PCR reaction with *amdat* specific primers. The PCR products were purified utilising Agencourt® AMPure® XP PCR Purification system (Beckman Coulter).

NGS library preparation

Libraries were prepared from 500-600ng of each amplicon utilising the NEBNext® DNA Library Prep Master Mix for Illumina®, and NEBNext® Multiplex Oligos for Illumina® Index Primers Set 1 and Set2 (New England Biolabs). Size selection of adaptor ligated DNA was performed using Agencourt AMPure XP beads (Beckman Coulter), with the bead:DNA ratio of the first bead selection 0.9X, followed by a second bead selection with bead:DNA ratio at 0.2X. Each library was eluted in 30 µL of 0.1X TE, library size confirmed via agarose gel electrophoresis, and diluted to a final concentration of 4nM.

NGS MiSeq sequencing

Next generation sequencing was performed on Illumina MiSeq instrument using MiSeq Reagent Kit v3 (Illumina) and 600 cycles. PhiX spike was added at 5% concentration as recommended by Illumina for low-diversity libraries.

Genome-wide methylation analysis

A custom script was written to extract differential methylation data from whole-genome bisulfite sequencing results. Genomic scaffold coordinates of each methylated cytosine together with methylation level information from each bs-seq sample were combined in a table and sorted on position. Using a window of 500 nucleotides the table was searched for regions containing at least ten (10) mCpGs with an average difference of not less than fifty percent (50%) between two selected samples.

Analysis of bs-seq results

For each analysed sample the frequency at which a mCpG occurred was calculated across all reads using custom Python scripts and open-source software. The process comprised of two steps. In the first, pairs of reads with the 30 nucleotide sequence starting at position 4 matching exactly the last 30 nucleotides of the primers used for nested amplicon PCR were extracted from FASTQ files, aligned with *in silico* bisulfite-converted genomic template using MUSCLE 57, overlapping regions (if any) were proportionally truncated and, after removing all aligner-introduced gaps, both reads were combined into one continuous sequence and appended to a separate file for each amplicon and each library/sample. In addition, a quality filter was applied, rejecting all sequences shorter than 90% of the length of the template or containing in excess of 5% gaps. an adjustment was applied in relation to indel-containing SVs/alleles. In the second step, batches of sequences from the "extract" files were re-aligned with the template using MUSCLE (to eliminate any potential positional errors introduced by read indels), the aligned template sequence was used to calculate positional information of all the expected CpGs and SNPs, and the positional data were used to score methylation status [ie. 0 for T and 1 for C occurring at a CpG position] and extract SV data [ie. the nucleotide at a SNP position and the read length for indels] for each combined read pair. The data were next appended to a separate table

for each amplicon and each library/sample.Methylation density was calculated as the percentage of methylated CpG motifs found across the AmDAT amplicons.

Expression analysis

AmDAT transcripts levels were quantitated via RT-PCR. cDNA was synthesized from 2.5 µg of RNA using Maxima reverse transcriptase (Thermo Scientific), as per the manufacturer's protocol and amplified using a SYBR® green I based assay. All RT-PCR experiments were performed utilising the Applied Biosystems® StepOnePlus™ Real-Time PCR System. Gene expression was normalised against both CAM and TBP, and relative expression calculated utilising the $2^{-\Delta\Delta CT}$ method, as previously described before Wedd et al 2016.

Identification of the truncated splice variant of amdat

The truncated AmDAT isoform was first discovered by analysing 454 RNA-seq reads mapped to *Apis mellifera* v.2.0 assembly with BLAT aligner (mapping by S. Foret; BLAT reference: Kent WJ. BLAT - the BLAST-like alignment tool. Genome Res. 2002 Apr;12(4):656-64). One read from pooled brain and ovary mRNA sample (ID: SRR063948.233094) showed an unusual splicing pattern.

Details from NCBI SRA database

Bioproject: PRJNA51483

SRA Study: SRP003261

Title: *Apis mellifera* strain MA transcriptome sequencing project

Abstract: Transcriptome sequencing of Apis mellifera was performed by the Baylor College of Medicine (BCM-HGSC), using testes, antennae, larvae, embryo, and brain and ovary samples provided by the R. Maleszka lab. Reads were assembled into contigs using Newbler, and submitted to GenBank as Transcriptome Shotgun Assemblies (TSAs).

Experiment: SRX025531

Biosample: SAMN02138538

The isoform was later detected in numerous honeybee RNA-seq datasets by BLAST-ing a 60 nucleotide, isoform-specific probe against NCBI SRA database: https://www.ncbi.nlm.nih.gov/sra/

The datasets in which *amdatΔ3* was found are listed in Table S3

In situ hybridization

The truncated fragment of *amdat* cloned into BlueScript was purified, cut with BamHI and NotI and transcribed in vitro with Promega T3 and T7 RNA polymerases to generate DIG-labeled sense and antisense probes. For more details see (Maleszka et al., 2007).

Primer sequences

For cloning of the full-length AmDAT cDNA, with restriction site adaptors

GB15426_clone_F TCAGGATCCACCATGTCGTCGAGGGTGGTGAAGAA

GB15426_clone_R CGTTCTAGAACCGCACTGTCCTAGACTGGCT

For qPCR (F3 - AmDAT-specific, F4 -AmDATΔ-specific)

DAT-qF3	AAAGGTGCGATCACTTGCTG
DAT-qF4	GTGTTATAAAAATGGTGGCGGAATT
DAT-qR3	AACGAGGCGAAGAAGTACCT

<u>For bisulfite amplicons (F1/R1 - first round PCR, F2/R2 - nested PCR):</u>

<u>Intron 2:</u>

DATin2-F1	ATAGATATATATAAATTTTGTTAG
DATin2-R1	ACTATATATAAATTTAATTCAAAC
DATin2-F2	GCAGAATTCTATTTTATTTATTGTTTTGTTATG
DATin2-R2	CGCAAGCTTTTTAAAAAATTACAATTAAATTCC

<u>Intron 3</u>:

DATex3-F1	ATTAATTTGATTAATATTTGAATG
DATex3-R1	ATCTTTTATTTAATTAAATCATTC
DATex3-F2	GCAGAATTCAAATGTTGTGAATTGTGTTTATAG
DATex3-R2	CGCAAGCTTTAATTAAAAATCCTCCTCAAATTC
DATin3-F1	AAAGAATTATTTAGTTTTGAAATA
DATin3-R1	TCAAAAATTATCTCTTCAAATATC
DATin3-F2	GCAGAATTCATAGAGGTTAATAATTGTTGGTTA
DATin3-R2	CGCAAGCTTTATCCTTATATCCTACTTCCCAAC

<u>Exon 10-11</u>

DATex10-11-F1	GTTTTTATTTTTTTATTTGTTGG
DATex10-11-R1	CTTTCTTAAACATATTCAAATCAA
DATex10-11-F2	GCAGAATTCATTYGATGTTGTTTGTAGTTTTGG
DATex10-11-R2	CGCAAGCTTAATAATCTTATAAATTACAATACC

Cloning, transformation and transcription of AmDAT and AmDAT2

The coding sequence of *amdat* was amplified with Promega GoTaq polymerase (Pfu added at a final concentration of 0.2%) using specially designed primers (see Table S2 for details). Cycling conditions: initial denaturation at 95°C for 5min, followed by 40 cycles of 95°C for 15s, 65°C for 20s and 72°C for 2min, and finally 72°C for 5min. The *amdat* PCR product was purified by agarose gel electrophoresis, and the 1963bp fragment was isolated using a QIAGEN MinElute Gel Extraction Kit. The purified fragment was excised using a BamHI/XbaI double digest and inserted into the same sites of the oocyte expression vector pGEM-He-Juel. Plasmid product was transformed into XL1-Blue Competent Cells (Agilent Tech), which were plated onto non-selective LB plates. Recombinant clones were selected using electrophoretic analysis of plasmids extracted from patch-plated colonies and cultured in liquid LB. Plasmids purified using a QIAPrep Spin Miniprep Kit (QIAGEN) were sequenced by the AGRF unit in Brisbane using specially designed primers (see Table S3 for details).

Amplification of endogenous *amdat2* proved difficult. An artificial clone was thus produced by double digesting *amdat*-containing plasmid with BamHI and EcoRI, and replacing the excised fragment with a synthetic sequence missing the alternatively spliced exon. This synthetic sequence was produced by GeneScript and received cloned into pUC57 using BamHI and EcoRI sites.

Maleszka, J., Foret, S., Saint, R., and Maleszka, R. (2007). RNAi-induced phenotypes suggest a novel role for a chemosensory protein CSP5 in the development of embryonic integument in the honeybee (Apis mellifera). *Dev Genes Evol* 217**,** 189-196.
